# Microwave Assisted Synthesis of Antioxidant Dihydro-Pyrazole Hybrids as Possible Lipoxygenase Inhibitors

**DOI:** 10.3390/molecules30102224

**Published:** 2025-05-20

**Authors:** Stergiani-Chrysovalanti Peitzika, Eirini Tsiampakari, Eleni Pontiki

**Affiliations:** Laboratory of Pharmaceutical Chemistry, School of Pharmacy, Faculty of Health Sciences, Aristotle University of Thessaloniki, 54124 Thessaloniki, Greece; speit@pharm.auth.gr (S.-C.P.); eirinitsiamp27@gmail.com (E.T.)

**Keywords:** antioxidants, dibenzacetones, dihydro-pyrazoles, inflammation, lipoxygenase, docking studies

## Abstract

Free radicals and inflammation have pivotal role in various degenerative diseases like cancer, rheumatoid arthritis, diabetes, cardiovascular and neurodegenerative disorders. Pyrazoles possess a wide range of biological activities such as antifungal, antituberculosis, antimicrobial, antiviral, anti-inflammatory, anti-convulsant, anticancer etc. In this present study a series of dibenzalacetones and the corresponding pyrazole hybrids were designed through bioisosterism, synthesized and biologically evaluated to highlight the importance of the extended conjugated system and substitution to the anti-inflammatory and antioxidant activity. The synthesis of dibenzalacetones was achieved via Claisen-Schmidt reaction. The dihydro-pyrazoles were synthesized from the substituted dibenzacetones and phenylhydrazines, hydrazine and semicarbazide under microwave irradiation optimizing reaction conditions. The synthesized compounds were spectroscopically characterized and evaluated for their anti-lipid peroxidation (AAPH) activity, their interaction with the free radical DPPH and the inhibition of soybean LOX. The novel derivatives were studied in terms of their physicochemical properties. Many of the dihydro-pyrazoles showed potent antioxidant properties and significant inhibition of soybean lipoxygenase as a result of their physicochemical features. Compounds **4a** and **4b** presented the most potent anti-lipid peroxidation abilities (98% and 97%), whereas compounds **2d** and **2e** have proved to be the most potent lipoxygenase inhibitors with IC_50_ values 2.5 μM and 0.35 μM. Moreover, docking studies with soybean lipoxygenase highlight the interactions of the novel derivatives with the enzyme.

## 1. Introduction

Inflammation is a biological response of homeostasis to various harmful stimuli [1,2]. It is a defensive and survivor mechanism of the immune system [3], involving complex responses of inflammatory mediators and a strong accumulation of oxidative stress products and lipid mediators. The inflammatory process is strongly associated with the production of free radicals [4] leading to peroxides and other reactive oxygen species [5]. The implication of reactive oxygen species (ROS), e.g., superoxide anion, hydroxyl radical and hydrogen peroxide, in diseases associated with oxidative stress (e.g., cardiovascular diseases, coronary artery disease, inflammatory injury, cancer) has been extensively referred in the literature [6,7,8,9,10].

Inflammation is associated with various diseases like cancer, rheumatoid arthritis, diabetes, cardiovascular and neurodegenerative disorders [11,12]. Inflammatory cytokines and mediators such as phospholipids, arachidonic acid, leukotrienes, prostaglandins, tumor necrosis factor play crucial role in the above-mentioned pathways [13]. During inflammation arachidonic acid is metabolized to prostaglandins via cyclooxygenase and leukotrienes via lipoxygenase, the main enzymatic routes of the inflammatory process [14].

Lipoxygenases (LOXs) catalyze the deoxygenation of Polyunsaturated Fatty Acids (PUFA) containing a 1-cis,4-cis-pentadiene unit to form conjugated hydroperoxydienoic acids using atmospheric oxygen. In their active center they contain a non-heme iron atom essential for the enzymatic activity [15]. Six lipoxygenase isoforms exist 5-LOX, 8-LOX, 12A-LOX, 12B-LOX, 15A-LOX, 15B-LOX [16], among them 5-lipoxygenase (5-LOX) is the one strongly associated with the production of leukotrienes (LTs), the inflammatory mediators of arachidonic acid [17].

**Figure 1:**
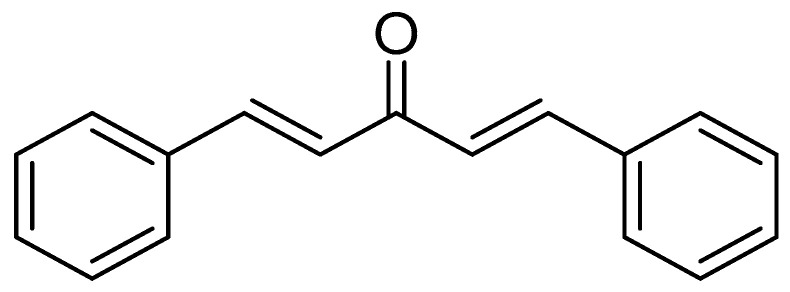
General structure of dibenzalacetones.

Dibenzalacetones have an aromatic ketone and an enone moiety and are an important component in a variety of biological compounds (Figure 1). Dibenzalacetones participate in a variety of chemical reactions and are intermediates in the synthesis of heterocyclic compounds such as, isoxazoles, quinolinones, thiadiazines and flavones [18]. They are also key intermediates in addition reactions such as cycloaddition and Michael addition due to the presence of enone functionality. In addition, dibenzalacetones show a wide range of biological activities [19], such as antifungal [20], antioxidant [21], antimalarial [22], analgesic [23] and anticancer [24,25].

**Figure 2:**
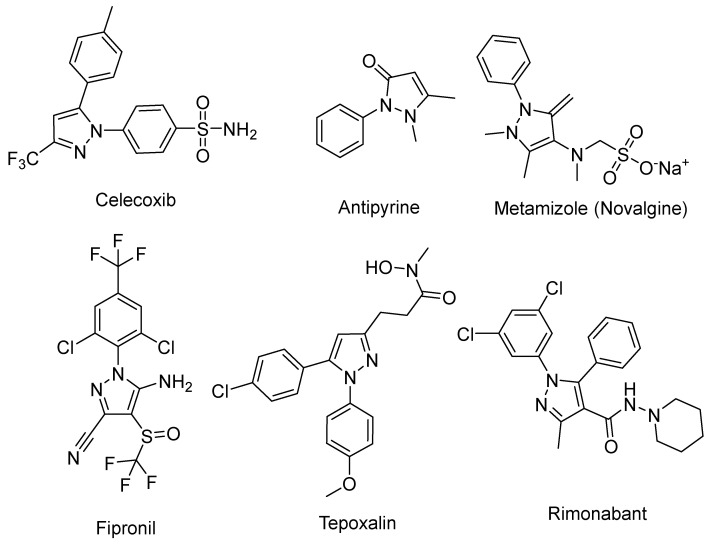
Drugs bearing the pyrazole scaffold.

Pyrazole is an aromatic heterocyclic system belonging to the azole class [26]. During the last decades, the development of synthetic strategies of pyrazoles and pyrazolines has gained much attention due to their biological applications. The most common synthetic methodology for pyrazoles and pyrazolines follows the cyclization of hydrazine with various electrophiles, including 1,3-dicarbonyl compounds, α,β-unsaturated carbonyl compounds and dihalides [27,28,29,30,31,32,33]. Pyrazoles are among the most important scaffolds possessing a wide variety of biological activities such as antifungal, antituberculosis, antimicrobial, antiviral, anti-inflammatory, anticonvulsant, anticancer and neuroprotective [34]. Furthermore, they act as inhibitors of the angiotensin-converting enzyme (ACE), as well as antagonists of the cholecystokinin receptor [34]. In addition, the pyrazole skeleton as well as pyrazoline are also associated with AChE inhibition and Alzheimer’s disease [35]. The privileged pyrazole scaffold is present in many drugs such as celecoxib (non steroidal anti-inflammatory drug), antipyrine (antipyretic), metamizole (painkiller, antipyretic and spasm reliever), fipronil (insecticide), tepoxalin (non steroidal anti-inflammatory drug used in veterinary medicine) and rimonabant (anorectic anti-obesity drug) (Figure 2).

A prerequisite in modern medicinal chemistry is the design and investigation of hybrid structures in order to optimize cost and efficiency [36]. The philosophy of hybrid drugs is the combination of two different pharmacophores or more in a single molecule, interacting simultaneously with multiple targets [37,38,39].The antiinflammatory activity of pyrazoles has been explored in recent literature using different protocols [40,41]. Moreover, Eid, N.M. and George, R.F. are focused on the synthesis of heterocyclic (methyl-substituted thienyl- and furyl-) curcumin derivatives [42]. The obtained dienones are converted into different pyrazolines in order to inverstigate the effect of these heterocyclic hybrids on the antiinflammatory activity. The results revealed promissing antiinflammatory activity for the new hybrids compared to indomethacin.

Based on the above study and in continuation of our group research work [40] and experience, a series of novel dihydro-pyrazole hybrids has been designed following bioisosteric replacements. The new hybrids were synthesized and evaluated for their antioxidant and lipoxygenase inhibitory activities in order to identify the crucial structural characteristics realated to the activity. The activities of dihydro-pyrazole hybrids were compared with the ones of the intermediate dibenzalacetones. Finally, the obtained novel compounds were subjected to molecular docking studies to define their potential interactions with soybean lipoxygenase.

## 2. Results

### 2.1. Chemistry

**Scheme 1:**
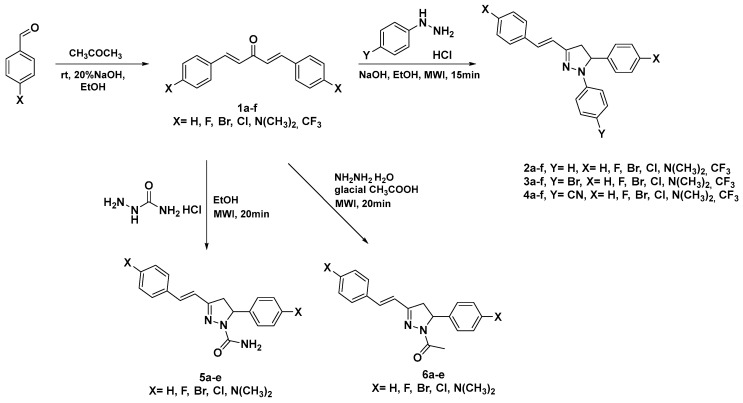
General Synthetic Scheme: Synthesis of dibenzalacetones (**1a**–**f**) using: abs. EtOH, aqueous NaOH 10% *w*/*v*, 25 °C, 1–24 h. Synthesis of dihydro-pyrazoles (**2a**–**f**, **3a**–**f**, **4a**–**f**) using: 4-substituted hydrazines, EtOH, NaOH, MW, 100 W, 75 °C, 15–70 min. Synthesis of pyrazole-carboxamides (**5a**–**e**) using: H_2_NNHC(=O)NH_2_, EtOH, MW, 200 W, 100 °C, 20 min. Synthesis of dihydro-pyrazol-ethanones (**6a**–**e**) using: N_2_H_4_·H_2_O, CH_3_COOH, MW, 200 W, 100 °C, 20 min.

Dihydropyrazoles (**2a**–**f**, **3a**–**f**, **4a**–**f**), pyrazole-carboxamides (**5a**–**e**) and dihydro-pyrazol-ethanones (**6a**–**e**) were synthesized from the corresponding dibenzalacetones (**1a**–**f**) as depicted in Scheme 1.The synthesis of dibenzalacetones (**1a**–**f**) was achieved via Claisen-Schmidt reaction [42]. For the synthesis of the novel hybrids (**2a**–**f**, **3a**–**f**, **4a**–**f**, **5a**–**e** and **6a**–**e**) microwave irradiation was applied (Scheme 1) optimizing the litterature conditions [42,43]. Novel derivatives were characterized by melting point, IR, ^1^H NMR, ^13^C NMR spectra and HRMS or elemental analysis.

Compounds (**1a**–**e**) were obtained in medium to good yields (30–90%). Compounds **1a**–**e** have been previously reported in the literature [44,45,46,47]. Compound **1f,** was confirmed by IR, ^13^C NMR spectra and elemental analysis. IR spectra revealed absorption bands at 1700–1650 cm^−1^ (C=O stretch) and 1600–1500 cm^−1^ (C=C bond stretch) while the absorption band of C=N bond appears stronger. In the ^1^H NMR spectra *trans*-vinyl hydrogens appear at *δ* 7.00–8.00 ppm as doublets (d), with *J-*coupling constant of 15.9 Hz, while the the remaining aromatic protons at *δ* 7.00–8.00 ppm. In the ^13^C NMR spectrum of compound **1f** all the carbons are recorded in the aromatic region i.e., at *δ* 120–150.

The dihydro-pyrazoles (**2a**–**f**, **3a**–**f**, **4a**–**f**) were obtained at yields 50–82%. Compounds **2a**–**d** and **3a** have been previously reported [43,48,49]. The structures of the novel derivatives were identified through ^1^H NMR, ^13^C NMR, IR and high-resolution mass spectrometry (HRMS). IR spectra presented the characteristic absorption bandsat 1600–1500 cm^−1^ (C=N and C=C bond stretch) and 1400–800 cm^−1^ (C-N bond stretch) [43,48,49]. The compounds bearing a CN-group present an additional absorption band at 2300–2000 cm^−1^. As for the ^1^H NMR data the proton of C5 of dihydropyrazole was recorded at *δ* 5.0 ppm, while the other two protons of C4 of dihydropyrazole were recorded slightly downfieldas a doublet of doublets (dd), due to the anisotropic effect of the chiral secondary carbon. The *trans-*vinyl hydrogens are recorded at *δ* 7.00–8.00 ppm as doublets (d), with *J*-coupling constant of 15.0–16.0 Hz. The ^13^C chemical shifts of the carbon atoms of dihydropyrazole are recorded at *δ* 43 (average value) for the -C4 and at *δ* 63 (average value) for the -C5 of dihydropyrazole at, while the aromatic ones at *δ* 100–165. In the HRMS spectra, the main ion presented was the [M + H]^+^.

The pyrazole-carboxamides (**5a**–**e**) and dihydro-pyrazol-ethanones (**6a**–**e**) were obtained at 8–51% yields. Compounds **5a**–**b**, **5e** and **6a**–**e** have been previously reported [50,51,52,53]. The novel derivatives were characterised through ^1^H NMR, ^13^C NMR, IR, melting point and elemental analysis. In accordance with the literature [50,51,52,53] IR spectra present absorption bands at 1600–1500 cm^−1^ (C=N, C=C bond stretch) and 1400–800 cm^−1^ (C-N bond stretch), while the pyrazole-carboxamides (**5c**–**d**) present absorption bands at 3500–3300 cm^−1^ (N-H stretch) 1690–1630 cm^−1^ (C=O stretch) and 1600–1500 cm^−1^ (C=C bond stretch). Regarding the ^1^H NMR spectra, the protons of NH_2_ and CH_3_ groups are recorded Finally, ^13^C NMR spectra present the characteristic C=O peak.

### 2.2. Physicochemical Studies

#### 2.2.1. Determination of Lipophilicity

Lipophilicity is a main physicochemical parameter for bioactive molecules, linking solubility, ligand-target binding interactions, membrane permeability absorption, distribution, bioavailability, metabolism, elimination and toxicological effects (ADMET), playing an important role in biological activity. The experimental values of lipophilicity R_M_ were determined by reverse phase thin layer chromatography. For the experimental determination of lipophilicity the hydrophilic-lipid phase system is a mixture of methanol- water (90/10%). The theoretical lipophilicity values are determined by the Bioloom program (http://biobyte.com/bb/prod/bioloom.html, accessed on 8 April 2024) (Table 1). According to the calculated clog *P* values, the most lipophilic compounds are **3c**, **3d** and **3f** while the less lipophilic ones are compounds **5a** and **5b**.

#### 2.2.2. Theoretical Calculation of Physicochemical Properties

A qualitative assessment of a molecule’s potential to be an oral drug in terms of bioavailability is called “drug-likeness”. Table 2 lists the results of drug-likeness study of the synthesized compounds using the molinspiration platform (https://www.molinspiration.com/ accessed on 8 April 2024). In particular, the values miLogP indicate lipophilicity, TPSA the topological polar surface area, Natoms the number of atoms in the molecule, MW the molecular weight, nON is the number of hydrogen bond acceptors, nOHNH the number of hydrogen bond donors, Nviolations o the number of violations of the rule of five, Nrotb o the number of rotated bonds and Volume the molecular volume. Perusal of the results show that compounds **1a**, **1b**, **1e**, **5a**, **5b**, **5e**, **6a**, **6b** do not present any violation. However, from the whole sum of 34 compounds only nine have drug likeness. The rest violate lipophilicity and/or MW. TPSA is frequently employed to estimate/predict cellular absorption of drugs and the optimal values ranges between 60–140 Å^2^. Drugs with TPSA values lower than 90 Å^2^ permeate the BBB.

### 2.3. Biological Evaluation

The synthesized compounds were evaluated in vitro for their ability to interact with the stable free radical DPPH, inhibit linoleic acid peroxidation induced by AAPH and soybean lipoxygenase (Table 3).

The ability of the derivatives to interact with the stable free 2,2-diphenyl-1-picrylhydrazyl radical (DPPH) at concentrations of 100 μM was examined after 20 and 60 min at 517 nm using nordihydroguaretic acid (NDGA) as reference compound. In general, the compounds present moderate to good antioxidant activity. It seems that the dibenzylacetones present satisfactory antioxidant activity compared to the hybrids at 60 min (**1b**, **1d** and **1f**). The existing activity of dibenzylacetones does not seem to be correlated with lipophilicity and molecular volume.

Compounds **2d** and **3d** show the best interaction among these series 45–53% at 60 min, while compounds **2a**, **2b**, **3a** and **3c** present moderate activity. Among the subgroup **2a**–**f** presenting a benzene ring as substituent at position 1 of the pyrazolyl ring, compound **2c** appears to be the most active. The presence of the dimethyl-amino group (**2e** and **3e**) generally contributed positively to the interaction with the DPPH. Among the subgroup of **3a**–**f** with the 4-bromobenzene substituent at position 1 of the pyrazolyl ring, the most active is compound **3e**, possessing the dimethyl-amino. Compounds **2e** and **3e** are the most active among all the derivatives. Compound **4e**, possessing a 4-cyanobenzyl substitution is almost inactive. Within the subgroup **4a**–**f** with the 4-cyanobenzyl substitution at position 1 of the pyrazolyl ring, the most active is the chloro-substituted, **4d**. For the majority of the compounds (**1a**, **1b**, **1d**, **6b**, **6e** and **5d**) the interaction seems to be increased over time. Dichalcone with a chloro-substituent at the 4-position shows an increased interaction over time. However, the transformation to the dihydro-pyrazol-ethanone (**6d**) and the pyrazole-carboxamide (**5d**) lead to a decrease of the antioxidant activity in relation to the starting material (dichalcone).

The compounds show lower interaction with the stable free radical and lower antioxidant ability at 60 min. Lipophilicity does not influence activity. However, the bulk of these compounds could prevent their interaction with the free radical.

The ability of the compounds to inhibit AAPH-induced peroxidation of linoleic acid is another assay to investigate the antioxidant properties of the compounds. Azo compounds such as 2,2′-azobis dihydrochloride (2-amidinopropane) (AAPH) are capable of generating free radicals through spontaneous decomposition at 37 °C. The R- radicals react immediately with air-oxygen to produce peroxy radicals [58]. AAPH was therefore used as the radical initiator and sodium linoleate as substrate subjected to peroxidation. Measurements were carried out at 234 nm using a U*V*/*V* is spectrophotometer. Dichalcone **1i** substituted with two phenyl groups presents the lowest lipophilicity. However, it shows the best activity (69%). Therefore, hydrophilic substituents offer an increase in activity.

The majority of compounds series **2a**–**f**, **3a**–**f**, **4a**–**f** seems to exhibit very good inhibition against lipid peroxidation apart from compound **4f** (12%). The most active compounds are **4a** (98%) and **4b** (97%) presenting better activity than the reference compound Trolox. These compounds present the lowest molecular refractivity within the series.

Lipophilicity seems to favor lipid peroxidation inhibitory activity. From series **2a**–**f** bearing the 1-phenylpyrazole group, compounds **2d** and **2e**, substituted with a chlorine and dimethyl-amino groups seem to be more active in the series. Among compounds **3a**–**f** possessing the 4-bromo-phenyl substituent at position 1 of the pyrazolyl ring, the most active are compounds **3e** and **3f**, with a dimethyl-amino and trifluoromethyl group respectively. In addition, from the 4-cyanosubstituted pyrazolines (**4a**–**f)** the most active are compounds **4a** and **4b**, substituted with a phenyl and 4-fluorinephenyl group, respectively. Among the phenyl substituted derivatives (**2a**, **3a**, **4a**) compounds **3a** and **4a** show the most pronounced inhibition of lipid peroxidation, and it appears that the phenyl substitution at position 1 of the heterocyclic ring leads to a rapid decrease in inhibitory activity (**2a**).

For the amide derivatives, the most significant inhibitory effect is exhibited by compound **5c**, which is the 4-bromo-pyrazoline amide derivative. Bromine has the higher lipophilic π-contribution. Thus, the lipophilic contribution of the substituent in position 4 highly influences the increase of the antioxidant activity. Furthermore, for acetyl-derivatives, compound **6c** (4-Br substituent) shows the best lipid peroxidation inhibition (71%). This effect is followed by compound **5c**. In general, the acetyl-pyrazoline derivatives show improved inhibitory activity compared to the original chalcones apart from compounds **1a** and **6a** respectively. However, none of the synthesized compounds present higher inhibition compared to the reference compound.

All compounds appear to show medium to very good inhibitory activity against lipid peroxidation with the only exception of compound **5b** (8%). Generally better activity is shown by compounds **4a** and **4b**.

Soybean lipoxygenase SLOX-1 at pH 9 is used in vitro. During this assay linoleic acid sodium salt is converted to the corresponding 13-hydroperoxide. The reaction is recorded photometrically at 234 nm using the UV-based enzyme assay [59]. This method does not provide quantitative results for the inhibition of mammalian 5-LOX. It is known that soybean LOX inhibitory activity induced by NSAIDs is qualitatively quite similar to the inhibition observed on the rat mast cells LOX and this assay may be used as a qualitative or semi-quantitative screen for such activity.

The best inhibition on SLOX is shown by the simple chalcone **1a** (IC_50_ = 10 μM). The other dichalcones show low inhibitory activity ranging between 24–38%. Among the members of series **2a**–**f** the most active compounds are **2d** (2.5 μM) and **2e** (0.35 μM) with a chlorine and dimethyl-amino substituent at 4-position respectively. The presence of electron donors substituents at the 4-position of the dibenzalketone groups influences the activity. The last one (**2e**) shows activity comparable to that of the reference compound NDGA (0.45 μM). For subgroup **3a**–**f** possessing the 4-bromo-benzyl substitution at position 1 of the pyrazolyl ring, the most active is compound **3v**, with a dimethyl-amino group. Similarly, among the compounds **4a**–**f** which have 4-cyano-benzyl substitution at position 1 of the pyrazole ring, the most active is compound **4d**, substituted with a chlorine. It is observed as in the case of antioxidant activity with the DPPH free radical, the most active compounds are those bearing the dimethylamino group, **2e** and **3e**. However, 4-cyano-substituted derivatives of dimethylamino-derived pyrazoles have appeared almost inactive **4e** (6%). Additionally, the compounds bearing chlorine in their structure, **2d** and **4d**, exhibit high inhibitory activity against LOX. However, replacing 4-chloro with 4-bromo-substitution does not seem to favor the activity, **3d** (11%).

Among the amide-derivatives the 4-chlorine substituted derivative (**5d**) presents the best inhibitory activity. Considering the acetyl pyrazoline derivatives, it appears that the presence of 4-dimethylamine substituent (**6e**) favors activity. Compound **6e**, with the best IC_50_ = 40 μM shows the highest substituent molecular refractivity (MR) value. It seems that the presence of a bulky substituent leads to an increase in inhibition. From the literature it is highlighted that MR of the substituents often contributes to lipoxygenase inhibitory activity, indicating favorable polar interactions [60]. Similarly, for the amide derivatives the 4-Cl substituted derivative shows the best activity (IC_50_ = 80 μM).

### 2.4. Docking Simulation Soybean Lipoxygenase Studies

Docking studies were performed to soybean lipoxygenase-1 (PDB ID: 3PZW) complying with the experimentally used protocol. Lipoxygenases are hyperoxidases containing a “non-heme” iron per molecule catalyzing the conversion of free and esterified polyunsaturated fatty acids to hydroperoxides. Crystal structure of soybean lipoxygenase-1 (PDB ID: 3PZW) lacks a co-crystallized ligand and imposes the identification of potential allosteric binding sites apart from the iron-binding site and the substrate-binding cavity as referred in recent literature. Detsi, A. et al. [61] have identified using SiteMap’s three potential binding sites [62]. According to researchers Site 1 is placed between the amino terminal β-barrel (PLAT domain) and the α-helical domain where the catalytic iron is located while Sites 2 and 3 are placed to the α-helical domain. Additionally, Site 1 has been previously referred as potential binding site when blind docking is accomplished [61,63,64].

**Figure 3:**
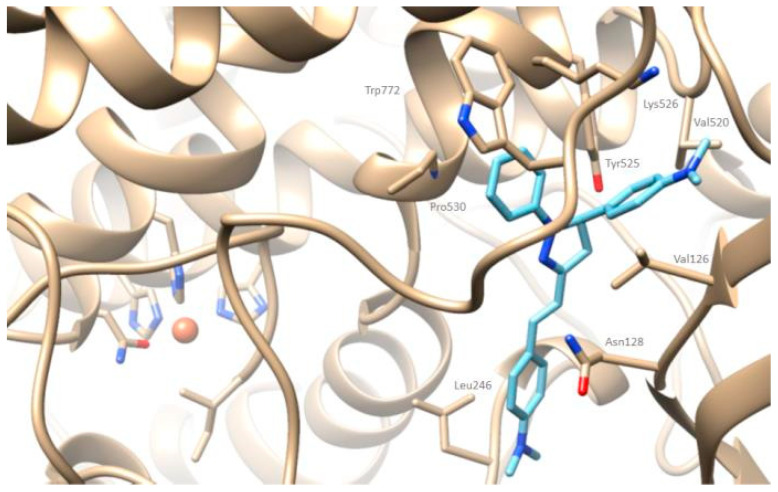
3D preferred docking pose of compound **2e** (depicted in cyan) bound to soybean lipoxygenase. Iron appears as an orange sphere.

**Figure 4:**
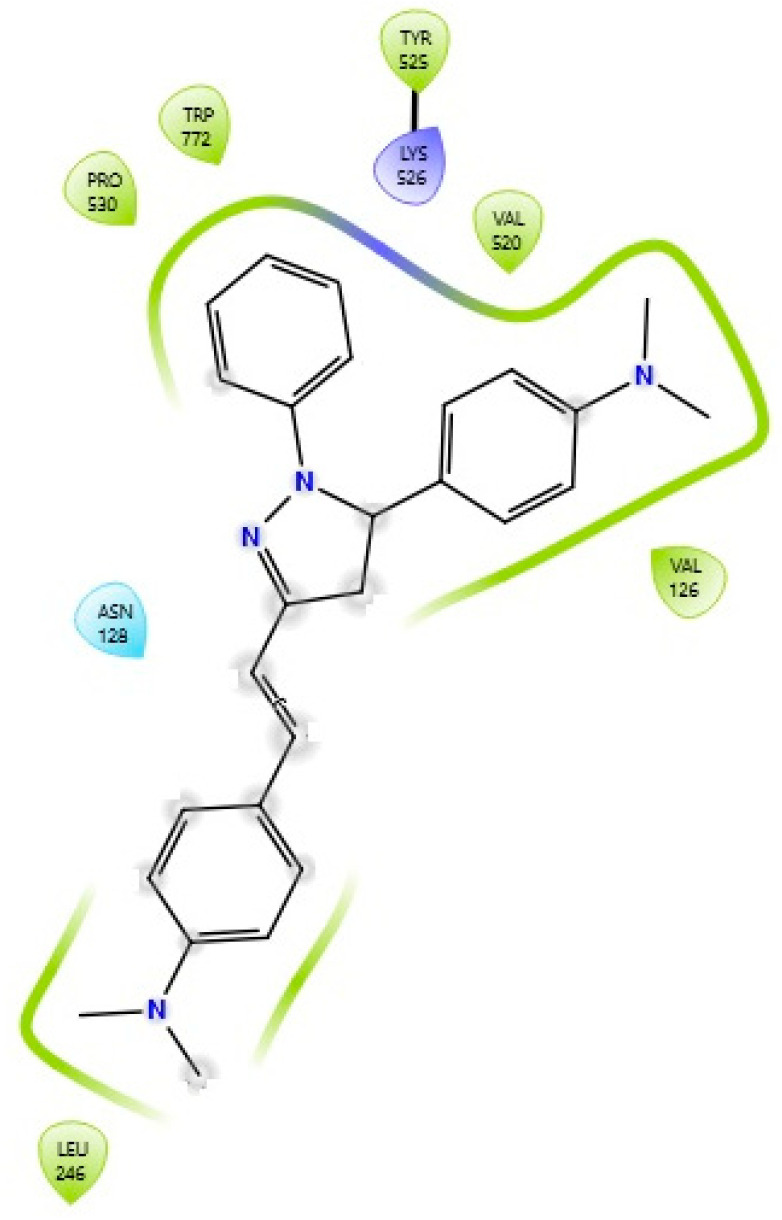
Ligand interaction diagram of compound **2e** with soybean lipoxygenase (ID: 3PZW). Hydrophobic residues are colored green, the polar ones cyan and the positive charged in blue. Figure made with free Maestro (Free Maestro academic license [65]).

Based on previous findings in this present study, compounds were evaluated for the mode of binding to both the active site as well as the whole enzyme with the aim of performing a comprehensive study of all possible allosteric centers. It appears that the compounds besides binding in an allosteric manner show similarities in terms of the binding mode. Probably due to stereochemical hindrances they are not allowed to enter the active site of the enzyme, which is confirmed by earlier studies. On the basis of the above results, it is concluded that the compounds act allosterically thereby blocking the enzyme activity. The most active compound **2e** presents a binding score of −9.7 kcal/mol and develops hydrophobic interactions with the amino acids Val126, Asn128, Leu246, Val520, Tyr525, Lys526, Pro530 and Trp772. Figure 3 shows the optimal binding mode of compound **2e** to the enzyme lipoxygenase from soybean (PDB ID: 3PZW). The iron of the active site is shown as an orange sphere. Moreover, Figure 4 describes the ligand interaction diagram of compound **2e** with soybean lipoxygenase (ID: 3PZW).

## 3. Experimental Section

### 3.1. Materials and Instruments

Analytical grade solvents, chemical and biochemical reagents have been used and purchased from commercial suppliers (Merck, Merck KgaA, Darmstadt, Germany, Fluka Sigma-Aldrich Laborchemikalien GmbH, Hannover, Germany, Alfa Aesar, Karlsruhe, Germany and Sigma, St. Louis, MO, USA). Soybean lipoxygenase, arachidonic acid (AA), 2,2-azinobis-2-methyl-propanimidamine HCl (AAPH) were obtained from Sigma Chemical, Co. Nordihydroguaretic acid (NDGA), 1,1-diphenyl-2-picrylhydrazyl (DPPH) and 6-Hydroxy-2,5,7,8-tetramethyl-chroman2-carboxylic acid (Trolox) were acquired from the Sigma-Aldrich Chemical Co. (St. Louis, MO, USA).

Melting points were determined on a MEL-Temp II (Lab. Devices, Holliston, MA, USA). In vitro tests were carried out using a UV-Vis spectra and recorded on a Perkin-Elmer 554 double-beam spectrophotometer (Perkin-Elmer Corporation Ltd., Lane Beaconsfield, Bucks, UK). Infrared spectra (KBr pellets) were recorded with Perkin-Elmer 597 spectrophotometer (Perkin-Elmer Corporation Ltd., Lane Beaconsfield, Bucks, UK). The ^1^H Nucleic Magnetic Resonance (^1^H-NMR) spectra were acquired on an Agilent 500/54 (DD2) at 500 MHz in CDCl_3_ using tetramethylsilane as an internal standard. Agilent 500/54 (DD2) ^13^C NMR spectra were acquired at 125 MHz in CDCl_3_ solutions with tetramethylsilane serving as an internal reference. Coupling constants *J* are expressed in Hz, and chemical shifts are reported in ppm. Thermo Fisher Scientific Inc., Waltham, MA, USA, used MeOH as the solvent to calculate mass spectra using an LC-PDA-MS Thermo Finnigan system (LC Pump Plus, Autosampler, Surveyor PDA Plus Detector) interfaced with an ESI MSQ Plus (Thermo Finnigan). Thin-layer chromatography using 5554 F254 Silica gel/TLC cards (Merck and Fluka Chemie GmbH Buchs, Steinheim, Switzerland) was used to monitor the reaction progress. Silica gel 60 F254 plates with a diameter of 2 mm were utilized for preparative thin layer chromatography (prep TLC) by Merck KgaA (ICH078057). Merck (20 × 20 cm) plates were used for the experimental assessment of the lipophilicity using reverse phase thin-layer chromatography (RP-TLC) TLC-Silica gel 60 F254 DC Kieselgel.

### 3.2. Chemistry General Procedure

#### 3.2.1. Synthesis of Dibenzalacetones (**1a**–**f**)

The appropriate substituted benzaldehyde (0.02 mol) and acetone (0.01 mol) were dissolved in absolute ethanol (16 mL) [42]. After stirring, sodium hydroxide solution (10% *w*/*v*) is added dropwise with cooling till the pH of the solution, becomes alkaline. The mixture is stirred at room temperature for 1–24 h, monitoring the reaction with thin layer chromatography (TLC). After completion of the reaction, the formed precipitate was filtered, washed with water, dried and recrystallized from ethanol/water or abs. ethanol to obtain the final products. With the exception of compound **1f,** all the others synthesized dibenzalacetones have been reported in the literature [44,45,46,47]. The identification of the compounds (**1a**–**e**) was carried out by the determination of the melting point and spectroscopic analysis through ^1^H NMR being in accordance with the literature [44,45,46,47].

(*1E*,*4E*)-1,5-diphenyl-1,4-pentadien-3-one (**1a**): The spectral data were in agreement with the literature data [44].

(*1E*,*4E*)-1,5-bis(4-fluorophenyl)-1,4-pentadien-3-one (**1b**): The spectral data were in agreement with the literature data [45].

(*1E*,*4E*)-1,5-bis(4-bromophenyl)-1,4-pentadien-3-oneone (**1c**): The spectral data were in agreement with the literature data [46].

(*1E*,*4E*)-1,5-bis(4-chlorophenyl)-1,4-pentadien-3-one (**1d**): The spectral data were in agreement with the literature data [46].

(*1E*,*4E*)-1,5-bis(4-dimethylamino phenyl)-1,4-pentadien-3-one (**1e**): The spectral data were in agreement with the literature data [47].

(*1E*,*4E*)-1,5-bis(4-(trifluoromethyl)phenyl)-1,4-pentadien-3-one (**1f**): Yield: 30%, yellowish solid, m.p.: 150–152 °C (recrystallisation from abs. C_2_H_5_OH). ^1^H NMR (500 MHz, CDCl_3_) δ 7.76 (d, *J* = 16.1 Hz, 2H, -C*H*=CHC(=O)CH=C*H*-), 7.73–7.67 (m, 8H, ArH), 7.14 (d, *J* = 15.9 Hz, 2H, -CH=C*H*C(=O)C*H*=CH-). ^13^C NMR (126 MHz, CDCl_3_): δ 188.2, 142.0, 138.1, 128.7, 127.3, 126.1 (q, *J* = 3.8 Hz), 123.9 (d, *J* = 271.3 Hz). IR (KBr, cm-1): 3400.6, 1655.9 (C=O), 1600.0 (C=C), 1320.3. Anal. C, H, N. Calc%: (C_19_H_12_F_6_O) C: 61.63, H: 3.27 Found%: C: 61.78, H: 3.36.

#### 3.2.2. Synthesis of Dihydro-Pyrazoles (**2a**–**f**, **3a**–**f**, **4a**–**f**)

The litterature synthetic method [43] of the dihydro-pyrazoles was optimised using microwave irradation.

Equimolar amounts of the appropriate substituted dibenzelacetones and 4-substituted phenylhydrazine hydrochloride (1 mmol) in absolute ethanol (3 mL) are added in a microwave vial. Sodium hydroxide (2.5 mmol, 0.10 g) is then added and the pH of the solution urned to alkaline. The resulting micture was irradiated at 100 W, at 75 °C, for 30 min, monitoring the reactionby thin layer chromatography (TLC). The formed precipitate is filtered under vacuum, washed with water and cold ethanol, dried and recrystalised from absolute ethanol or ethanol-water or ethyl acetate and petroleum ether to obtain the final products **2a**–**f**, **3a**–**f**, **4a**–**f** in pure form. Some of the synthesized pyrazoles have been reported in the literature [43,48,49,50,66].

(*E*)-1,5-Diphenyl-3-styryl-4,5-dihydro-1H-pyrazole (**2a**): The spectral data were in agreement with the literature data [43].

(*E*)-5-(4-fluorophenyl)-3-(4-(fluorostyryl)-1-phenyl-4,5-dihydro-1H-pyrazole (**2b**): The spectral data were in agreement with the literature data [48].

(*E*)-5-(4-bromophenyl)-3-(4-(bromostyryl)-1-phenyl-4,5-dihydro-1H-pyrazole (**2c**): The spectral data were in agreement with the literature data [49].

(*E*)-5-(4-chlorophenyl)-3-(4-(chlorostyryl)-1-phenyl-4,5-dihydro-1H-pyrazole (**2d**): The spectral data were in agreement with the literature data [49].

(*E*)-4-[2-(5-(4-dimethylamino)phenyl)]-1-phenyl-4,5-dihydro-(1H-pyrazol-3-yl)-vinyl)-N,N-dimethylaniline **(2e)**: The spectral data were in agreement with the literature data [43].

(*E*)-1-phenyl-5-[(4-trifluoromethyl)-phenyl)]-3-[(4-trifluoromethyl)-styryl]-4,5-dihydro-1H-pyrazole (**2f**): Yield: 50%, yellow solid, m.p.: 188–190 °C (recrystallisation from C_2_H_5_OH/H_2_O). ^1^H NMR (500 MHz, CDCl_3_): δ 7.60 (t, *J* = 8.8 Hz, 3H, ArH), 7.53 (d, *J* = 8.2 Hz, 2H, ArH), 7.41 (d, *J* = 8.0 Hz, 2H), 7.30 (d, *J* = 16.3 Hz, 1H, -C*H*=CH-), 7.25–7.16 (m, 3H, ArH), 7.02–6.96 (m, 2H, ArH), 6.87–6.80 (m, 1H, ArH), 6.54 (d, *J* = 16.3 Hz, 1H, -CH=C*H*-), 5.36 (dd, *J* = 12.4, 6.6 Hz, 1H, -C*H*-, C5 of dihydropyrazole), 3.76 (dd, *J* = 16.7, 12.4 Hz, 1H, -CH_2_-, C4 of dihydropyrazole), 3.01 (dd, *J* = 16.8, 6.6 Hz, 1H, -CH_2_-, C4 of dihydropyrazole). ^13^C NMR (126 MHz, CDCl3): δ 147.7, 146.1, 143.7, 140.1, 131.1, 130.0, 129.7, 129.3, 126.5 (q, *J* = 3.8 Hz), 126.8, 126.3, 125.9 (q, *J* =3.8 Hz), 124.7 (d, *J* = 280 Hz), 123.9, 120.2, 113.6. IR (KBr, cm−1): 1594.4 (C=N), 1499.3 (C=C), 1320.3 (C-N), 1225.2, HRMS (MeOH) (ESI, *m*/*z*), (M.W. 460.1374) [M+H]^+^ = 461.11418.

(*E*)-1-(4-bromophenyl)-5-phenyl-3-styryl-4,5-dihydro-1H-pyrazole (**3a**): The spectral data were in agreement with the literature data [42].

(*E*)-1-(4-bromophenyl)-5-(4-fluorophenyl)-3-(4-fluorophenyl)-4,5-dihydro-1H-pyrazole (**3b**): Yield: 80%, orange solid, m.p.: 154–156 °C (recrystallisation from CH_3_CO_2_CH_2_CH_3_/petroleum ether). ^1^H NMR (500 MHz, CDCl_3_): δ 7.41 (dd, *J* = 8.9, 5.3 Hz, 2H, ArH), 7.27–7.19 (m, 2H, ArH), 7.10–7.12 (m, 2H, ArH), 7.09 (d, *J* = 16.4 Hz, 1H, -C*H*=CH-), 7.01–7.06 (m, 4H, ArH), 6.90 (d, *J* = 9.0 Hz, 2H, ArH), 6.52 (d*, J* = 16.4 Hz, 1H, -CH=C*H*-), 5.21 (dd, *J* = 12.2, 6.6 Hz, 1H, -C*H*-, C5 of dihydropyrazole), 3.70 (dd, *J* = 16.8, 12.2 Hz, 1H, -CH_2_-, C4 of dihydropyrazole), 2.98 (dd, *J* = 16.8, 6.6 Hz, 1H, -CH_2_-, C4 of dihydropyrazole). ^13^C NMR (126 MHz, CDCl_3_): δ 162.8 (d, *J* = 247.5 Hz), 162.4 (d, *J* = 245 Hz), 148.8, 142.6, 137.7 (d, *J* = 3.2 Hz), 132.8 (d, *J* = 3.1 Hz), 132.1, 129.0, 128.3 (d, *J* = 8.1 Hz), 127.6 (d, *J* = 8.1 Hz), 124.5, 121.2 (d, *J* = 2.6 Hz), 116.4 (d, *J* = 21.7 Hz), 114.7, 63.6, 42.5. IR (KBr, cm^−1^): 1594.4 (C=N), 1504.9 (C=C), 1328.7 (C-N), 1233.6., HRMS (MeOH) (ESI, *m*/*z*), (M.W. 438.0543) [M-147.0484 + K + 2MeOH]^+^ = 395.1118.

(*E*)-1-(4-bromophenyl)-5-(4-bromophenyl)-3-(4-bromophenyl)-4,5-dihydro-1H-pyrazole (**3c**): Yield: 52%, yellow solid, m.p.: 212–214 °C (recrystallisation from CH_3_CO_2_CH_2_CH_3_/petroleum ether). ^1^H NMR (500 MHz, CDCl_3_): δ 7.50–7.43 (m, 4H, ArH), 7.33–7.24 (m, 2H, ArH), 7.16 (d*, J* = 16.3 Hz, 1H, -C*H*=CH-), 7.12 (dd, *J* = 8.8, 2.8 Hz, 4H, ArH), 6.92–6.85 (m, 2H, ArH), 6.47 (d, *J* = 16.3 Hz, 1H, -CH=C*H*-), 5.20 (dd, *J* = 12.3, 6.6 Hz, 1H, -C*H*-, C5 of dihydropyrazole), 3.70 (dd, *J* = 16.8, 12.3 Hz, 1H, -CH_2_-, C4 of dihydropyrazole), 2.98 (dd, *J* = 16.8, 6.5 Hz, 1H, C4 of dihydropyrazole).^13^C NMR (126 MHz, CDCl_3_): δ 151.4, 143.3, 135.9, 132.1, 132.0, 130.3, 130.2, 129.5, 128.2, 126.3, 121.9, 120.7, 105.7, 55.6, 40.6. IR (KBr, cm^−1^): 1591.6 (C=N), 1490.9 (C=C), 1320.3 (C-N). HRMS (MeOH) (ESI, *m*/*z*), (M.W. 557.8941) [M-195.9636 + Na]^+^ = 385.0917.

(*E*)-1-(4-bromo-phenyl)-5-(4-chloro-phenyl)-3-(4-chlorostyryl)-4,5-dihydro-1H-pyrazole (**3d**): Yield: 68%, yellowish solid, m.p.: 190–192 °C (recrystallisation from CH_3_CO_2_CH_2_CH_3_/petroleum ether). ^1^H NMR (500 MHz, CDCl_3_): δ 7.36 (d, *J* = 8.6 Hz, 2H, ArH), 7.34–7.28 (m, 4H, ArH), 7.21–7.14 (m, 3H, -C*H*=CH- and 2H ArH), 7.17–7.08 (m, 2H, ArH), 6.92–6.85 (m, 2H, ArH), 6.49 (d, *J* = 16.3 Hz, 1H, -C*H*=CH-), 5.21 (dd, *J* = 12.3, 6.6 Hz, 1H, -C*H*-, C5 of dihydropyrazole), 3.70 (dd, *J* = 16.8, 12.3 Hz, 1H, -CH_2_-, C4 of dihydropyrazole), 2.97 (dd*, J* = 16.8, 6.6 Hz, 1H, -CH_2_-, C4 of dihydropyrazole). ^13^C NMR (126 MHz, CDCl_3_): δ 148.6, 142.5, 140.3, 135.1, 134.1, 133.8, 132.0, 129.6, 129.2, 129.1, 127.9, 127.3, 124.6, 121.9, 114.7, 63.7, 42.3. IR (KBr, cm^−1^): 1591.6 (C=N), 1490.9 (C=C), 1400.0 (C-N), 1320.3. HRMS (MeOH) (ESI, *m*/*z*), (M.W. 469.9952) [M-154.9496 + MeOH + K]^+^ = 385.1446.

(*E*)-4-(1-(4-bromophenyl))-3-(4-dimethylamino)-styryl-4,5-dihydro-(1H-pyrazol-3-yl)-vinyl)-N,N-dimethylaniline (**3e**): Yield: 52%, orange solid, m.p.: 215–217 °C (recrystallisation from CH_3_CO_2_CH_2_CH_3_/petroleum ether). ^1^H NMR (500 MHz, CDCl_3_): δ 7.36–7.33 (m, 2H, ArH), 7.13–7.08 (m, 4H, ArH), 6.98–7.03 (m, 1H, ArH), 6.96–6.93 (m, 2H -C*H*=CH- and ArH), 6.65–6.70 (m, 4H, ArH), 6.50 (d, *J* = 16.3 Hz, 1H, C*H*=CH-), 5.09 (ddd, *J* = 10.8, 12.6, 3.4 Hz, 1H, -CH_2_-, C4 of dihydropyrazole), 3.64 (ddd, *J* = 16.0, 11.9, 3.5 Hz, 1H, -CH_2_-, C4 of dihydropyrazole), 2.99 (s, 7H, -CH_2_-, C4 of dihydropyrazole and 2 CH_3_ groups) 2.93 (s, 6H, 2 CH_3_ groups). ^13^C NMR (126 MHz, CDCl_3_): δ 150.5, 150.1, 143.4, 133.7, 129.8, 128.8, 128.0, 126.8, 125.0, 123.4, 117.3, 114.5, 113.0, 112.4, 63.8, 42.8, 40.7, 40.5. IR (KBr, cm^−1^): 1594.4 (C=N), 1493.7 (C=C), 1356.6 (C-N), 1328.7. HRMS (MeOH) (ESI, *m*/*z*), (M.W. 488.1576) [M-147.0922 + 2MeOH + K + H]^+^ = 445.0893.

(*E*)-1-(4-bromophenyl)-5-[(4-trifluoromethyl)-phenyl)]-3-[(4-trifluoromethyl)-styryl]-4,5-dihydro-1H-pyrazole (**3f**): Yield: 52%, yellow solid, m.p.: 177–179 °C (recrystallisation from C_2_H_5_OH/H_2_O). ^1^H NMR (500 MHz, CDCl_3_): δ 7.65–7.57 (m, 4H, ArH), 7.53 (dd, *J* = 8.5, 3.0 Hz, 2H, ArH), 7.38 (dd, *J* = 8.4, 3.1 Hz, 2H, ArH), 7.27 (d, *J* = 16.4 Hz, 1H, -CH=C*H*-), 7.18–7.11 (m, 2H, ArH), 6.89–6.92 (m, 2H), 6.56 (d, *J* = 16.4 Hz, 1H, -C*H*=CH-), 5.33 (dd, *J* = 12.3, 6.7 Hz, 1H, -C*H*-, C5 of dihydropyrazole), 3.77 (dd, *J* = 16.8, 12.3 Hz, 1H, -CH_2_-, C4 of dihydropyrazole), 3.02 (dd, *J* = 16.8, 6.6 Hz, 1H, -CH_2_-, C4 of dihydropyrazole). ^13^C NMR (126 MHz, CDCl_3_): δ 148.1, 145.5, 142.0, 139.8, 131.5, 129.1, 126.7, 126.4 (q, *J* = 3.8 Hz), 126.1, 125.8 (q, *J* = 3.8 Hz), 124.9, 123.5, 114.6, 63.7, 42.0. IR (KBr, cm^−1^): 1594.4 (C=N), 1493.7 (C=C), 1398.8 (C-N), 1323.1. HRMS (MeOH) (ESI, *m*/*z*), (M.W. 538.0479) [M + K]^+^ = 577.1337

(*E*)-4-(5-phenyl-3-styryl-4,5-dihydro-1H-pyrazo-1-yl)-benzonitrile (**4a**): The spectral data were in agreement with the literature data [42].

(*E*)-4-(5-(4-fluorophenyl)-3-(4-fluorostyryl)-4,5-dihydro-1H-pyrazo-1-yl)-benzonitrile (**4b**): Yield: 73%, yellow solid, m.p.: 228–230 °C (recrystallisation from C_2_H_5_OH). ^1^H NMR (500 MHz, CDCl_3_): δ 7.44–7.40 (m, 4H, ArH), 7.21–7.17 (m, 2H, ArH), 7.10 (d, *J* = 16.4 Hz, 1H, -CH=C*H*-), 7.02–7.07 (m, H), 6.97 (d, *J* = 8.5 Hz, 2H), 6.60 (d, *J* = 16.3 Hz, 1H, -C*H*=CH-), 5.30 (dd, *J* = 12.1, 5.5 Hz, 1H, -C*H*-, C5 of dihydropyrazole), 3.75 (dd, *J* = 17.0, 12.1 Hz, 1H, -CH_2_-, C4 of dihydropyrazole), 3.04 (dd, *J* = 16.9, 5.5 Hz, 1H, -CH_2_-, C4 of dihydropyrazole). ^13^C NMR (126 MHz, CDCl_3_): δ 150.9, 146.4, 141.6, 136.8, 133.8, 133.5, 132.4, 128.6 (d, *J =* 8.1 Hz), 127.4 (d*, J* = 8.1 Hz), 120.7, 120.2, 116.6 (d, *J =* 21.8 Hz), 116.1 (d, *J =* 21.8 Hz), 113.2, 101.1 62.8, 42.5. IR (KBr, cm^−1^): 2212.9 (C≡N), 1597.2 (C=N), 1507.7 (C=C), 1339.9 (C-N). HRMS (MeOH) (ESI, *m*/*z*) (M.W. 385.1391) [M + H]^+^ = 386.14546.

(*E*)-4-(5-(4-bromophenyl)-3-(4-bromostyryl)-4,5-dihydro-1H-pyrazol-1-yl)-benzonitrile (**4c**): Yield: 78%, yellow solid, m.p.: 250–252 °C (recrystallisation from CH_3_CO_2_CH_2_CH_3_/petroleum ether). ^1^H NMR (500 MHz, CDCl_3_): δ 7.48 (dd, *J* = 8.4, 1.6 Hz, 4H, ArH), 7.42 (d, *J* = 9.0 Hz, 2H, ArH), 7.31 (d, *J* = 8.6 Hz, 2H, ArH), 7.16 (d, *J* = 16.4 Hz, 1H, -CH=C*H*-), 7.09 (d, *J* = 8.4 Hz, 2H, ArH), 6.96 (d, *J* = 8.8 Hz, 2H, ArH), 6.56 (d, *J* = 16.4 Hz, 1H, -C*H*=CH-), 5.28 (dd, *J* = 12.2, 5.6 Hz, 1H, -C*H*-, C5 of dihydropyrazole), 3.75 (dd, *J* = 17.0, 12.2 Hz, 1H, -CH_2_-, C4 of dihydropyrazole), 3.04 (dd, *J* = 17.1, 5.6 Hz, 1H, -CH_2_-, C4 of dihydropyrazole). ^13^C NMR (126 MHz, CDCl_3_): δ 150.7, 146.2, 135.1, 133.7, 133.5, 132.8, 132.2, 128.3, 127.4, 121.5, 113.2, 101.4, 62.9, 42.3. IR (KBr, cm^−1^): 2212.9 (C≡N), 1600.0 (C=N), 1513.3 (C=C), 1328.7 (C-N). HRMS (MeOH) (ESI, *m*/*z*), (M.W. 504.9789) [M + H]^+^ = 505.9847.

(*E*)-4-(5-(4-chlorophenyl)-3-(4-chlorostyryl)-4,5-dihydro-1H-pyrazol-1-yl)-benzonitrile (**4d**): Yield: 75%, yellow solid, m.p.: 227–229 °C (recrystallisation from CH_3_CO_2_CH_2_CH_3_/petroleum ether). ^1^H NMR (500 MHz, CDCl_3_): δ 7.42 (d, *J* = 8.6 Hz, 2H, ArH), 7.38 (d, *J* = 8.6 Hz, 2H, ArH), 7.36–7.30 (m, 3H, -CH=C*H*- and 2H ArH), 7.18–7.12 (m, 3H, ArH), 6.97 (d, *J* = 8.6 Hz, 2H, ArH), 6.58 (d, *J* = 16.3 Hz, 1H, -C*H*=CH-), 5.30 (dd, *J* = 12.2, 5.5 Hz, 1H, -C*H*-, C5 of dihydropyrazole), 3.75 (dd, *J* = 17.0, 12.2 Hz, 1H, -CH_2_-, C4 of dihydropyrazole), 3.04 (dd, *J* = 17.0, 5.5 Hz, 1H, -CH_2_-, C4 of dihydropyrazole). ^13^C NMR (126 MHz, CDCl_3_): δ 147.7, 146.2, 143.7, 140.1, 131.1, 129.7, 129.3, 126.8, 126.5, 126.3, 125.9, 124.0, 120.2, 113.7, 63.9, 42.1. IR (KBr, cm^−1^): 2218.5 (C≡N), 1600.0 (C=N), 1510.5 (C=C), 1328.7 (C-N). HRMS (MeOH) (ESI, *m*/*z*), (M.W. 417.0800) [M + H]^+^ = 418.0863.

(*E*)-4-(5-(4-(dimethylamino)phenyl)-3-(4-(dimethylamino)styryl)-4,5-dihydro-1H-pyrazo-1yl)-benzonitrile (**4e**): Yield: 55%, orange solid, m.p.: 220–222 °C (recrystallisation from CH_3_CO_2_CH_2_CH_3_/petroleum ether). ^1^H NMR (500 MHz, CDCl_3_): δ 7.36 (dd, *J* = 14.9, 9.0 Hz, 4H, ArH), 7.07 (d, *J* = 8.9 Hz, 2H, ArH), 7.02–6.96 (m, 3H, -CH=C*H*- and 2H ArH), 6.69–6.65 (m, 4H, ArH), 6.57 (d, *J* = 16.3 Hz, 1H, -C*H*=CH-), 5.18 (dd, *J* = 11.9, 5.4 Hz, 1H, -C*H*-, C5 of dihydropyrazole), 3.68 (dd, *J* = 17.5, 11.9 Hz, 1H, -CH_2_-, C4 of dihydropyrazole), 3.05 (dd, *J* = 16.8, 5.5 Hz, 1H, -CH_2_-, C4 of dihydropyrazole), 3.00 (s, 6H, 2 CH_3_ groups), 2.92 (s, 6H, 2 CH_3_ groups). ^13^C NMR (126 MHz, CDCl_3_): δ 152.4, 150.7, 146.8, 135.4, 133.3, 130.5, 128.2, 126.6, 124.4, 120.7, 116.5, 113.0, 112.9, 112.3, 99.7, 62.8, 42.7, 40.6, 40.4. IR (KBr, cm^−1^): 2207.3 (C≡N), 1597.20 (C=N), 1516.1 (C=C), 1334.3 (C-N). HRMS (MeOH) (ESI, *m*/*z*), (M.W. 435.2423) [M + H]^+^ = 436.2493.

(*E*)-4-(5-(4-(trifluoromethyl)phenyl)-3-(4-(trifluoromethyl)styryl)-4,5-dihydro-1H-pyrazol-1-yl)-benzonitrile (**4f**): Yield: 67%, yellowish solid, m.p.: 245–247 °C (recrystallisation from C_2_H_5_OH). ^1^H NMR (500 MHz, CDCl_3_): δ ^1^H NMR (500 MHz, CDCl_3_): δ 7.66–7.59 (m, 4H, ArH), 7.54 (d, *J* = 8.2 Hz, 2H, ArH), 7.44 (d, *J* = 8.8 Hz, 2H, ArH), 7.35 (d, *J* = 8.1 Hz, 2H, ArH), 7.27 (d, *J* = 16.4 Hz, 1H, -CH=C*H*-), 6.98 (d, *J* = 8.9 Hz, 2H, ArH), 6.64 (d, *J* = 16.4 Hz, 1H, -C*H*=CH-), 5.41 (dd, *J* = 12.3, 5.5 Hz, 1H, -C*H*-, C5 of dihydropyrazole), 3.82 (dd, *J* = 17.0, 12.3 Hz, 1H, -CH_2_-, C4 of dihydropyrazole), 3.08 (dd, *J* = 17.0, 5.5 Hz, 1H, -CH_2_-, C4 of dihydropyrazole). ^13^C NMR (126 MHz, CDCl_3_): δ 150.3, 146.1, 144.8, 139.5, 133.6, 133.3, 127.0, 126.8 (q, *J* = 3.8 Hz), 126.1, 126.0 (q, *J* = 3.8 Hz), 123.1, 120.0, 113.3, 63.1, 42.2. IR (KBr, cm^−1^): 2212.9 (C≡N), 1602.8 (C=N), 1513.3 (C=C), 1328.7 (C-N). HRMS (MeOH) (ESI, *m*/*z*), (M.W. 485.1327) [M + H]^+^ = 486.1395.

#### 3.2.3. Synthesis of Pyrazole-Carboxamides (**5a**–**e**)

To a vial suitable for microwave irradiation (Microwave Irradiation MWI) were added 0.5 mmol of the corresponding dibenzalacetone and 0.55 mmol of semicarbazide hydrochloride. Absolute ethanol (1.25 mL) is used as solvent. The reaction is irradiated at 200 W, at 75 °C for 20 min. The progress of the reaction is monitored by thin-layer chromatography (TLC). When the reaction is completed, the reaction mixture is decanted into a beaker containing cold deionized water and ice. A precipitate is formed which is collected by filtration under vacuum, washed with deionized water, dried and recrystallized from methanol/water or purified through preparative layer chromatography to give the final product.

(*E*)-5-phenyl-3-styryl-4,5-dihydro-1H-pyrazole-1-carboxamide (**5a**): The spectral data were in agreement with the literature data [50].

(*E*)-5-(4-fluorophenyl)-3-(4-fluorostyryl)-4,5-dihydro-1H-pyrazole-1-carboxamide (**5b**): Yield: 9%, m.p.: 97–99 °C (PLC 5/95 *v*/*v* CH_3_OH/CHCl_3_). ^1^H NMR (500 MHz, CDCl_3_): δ ^1^H NMR (500 MHz, CDCl_3_): δ 7.43 (dd, *J* = 8.9, 5.3 Hz, 2H, ArH), 7.20 (dd, *J* = 8.7, 5.2 Hz, 2H, ArH), 7.06 (t, *J* = 8.6 Hz, 2H, ArH), 7.01 (t, *J* = 8.7 Hz, 2H, ArH), 6.96 (d, *J* = 16.4 Hz, 1H, -C*H*=CH-), 6.68 (d, *J* = 16.4 Hz, 1H, -C*H*=CH-), 5.45 (dd, *J* = 12.0, 5.0 Hz, 1H, -C*H*-, C5 of dihydropyrazole), 3.62 (dd, *J* = 17.3, 12.0 Hz, 1H, -CH_2_-, C4 of dihydropyrazole), 2.99 (dd, *J* = 17.3, 5.1 Hz, 1H, -CH_2_-, C4 of dihydropyrazole).^13^C NMR (125 MHz, CDCl_3_): δ 163.2 (d, *J* = 248.8 Hz), 162.3 (d, *J* = 245 Hz), 155.3, 152.9, 138.3 (d, *J* = 3.2 Hz), 135.6, 132.0 (d, *J* = 3.4 Hz), 128.8 (d, *J* = 8.1 Hz), 127.3 (d, *J* = 8.1 Hz), 120.4 (d, *J* = 2.4 Hz), 116.1 (d, *J* = 21.9 Hz), 115.9 (d, *J* = 21.6Hz), 59.5, 41.8. Anal. C, H, N. Calc%: (C_18_H_15_F_2_N_3_O) C: 66.05, H: 4.62, N: 12.84 Found%: C: 65.97, H: 4.57, N: 12.64.

(*E*)-5-(4-bromophenyl)-3-(4-bromostyryl)-4,5-dihydro-1H-pyrazole-1-carboxamide (**5c**): Yield: 15%, m.p.: 170–172 °C (recrystallisation from CH_3_OH/H_2_O). ^1^H NMR (500 MHz, Methanol-d4): δ ^1^H NMR (500 MHz, CD_3_OD) δ 7.82 (s, 1H), 7.63 (d, *J* = 8.3 Hz, 2H), 7.55 (d, *J* = 8.5 Hz, 2H), 7.52 (d, *J* = 8.5 Hz, 1H), 7.49 (d, *J* = 8.4 Hz, 1H), 7.46 (d, *J* = 8.3 Hz, 2H), 7.17 (d, *J* = 15.9 Hz, 1H), 7.14 (s, 1H), 6.86 (d, *J* = 16.5 Hz, 1H), 5.41 (dd, *J* = 12.0, 5.1 Hz, 1H), 3.72 (dd, *J* = 17.6, 12.1 Hz, 1H), 3.03–2.97 (m, 1H). ^13^C NMR (125 MHz, CDCl_3_): δ 171.6, 152.4, 135.3, 135.1, 132.1, 132.0, 131.9, 131.3, 130.2, 128.3, 127.2, 121.1, 59.6, 41.5. IR (KBr, cm^−1^): 3465.6 (N-H stretch), 1670.7 (C=O stretch), 1588.7 (C=C bond stretch), 1487.8, 1400.8. Anal. C, H, N. Calc%: (C_18_H_15_Br_2_N_3_O) C: 48.14, H: 3.37, N: 9.36 Found%: C: 48.19, H: 3.41, N: 9.39.

(*E*)-5-(4-chlorophenyl)-3-(4-chlorostyryl)-4,5-dihydro-1H-pyrazole-1-carboxamide (**5d**): The spectral data were in agreement with the literature data [44].

(*E*)-5-(4-dimethylaminophenyl)-3-(4-dimethylaminostyryl)-4,5-dihydro-1H-pyrazole-1-carboxamide (5e): The spectral data were in agreement with the literature data [53].

#### 3.2.4. Synthesis of Dihydro-Pyrazol-Ethanones (**6a**–**e**)

To a vial suitable for microwave irradiation were added 0.5 mmol of the corresponding dibenzalacetone and 1 mmol of 98% hydrazine monohydrate inglacial acetic acid (1.25 mL). The reaction is irradiated at 200 W, at 100 °C and for 20 min. The reaction is monitored by TLC. Upon completion, the reaction mixture is decanted into a beaker containing cold deionized water and ice. A precipitate is formed which is collected by filtration under vacuum, washed with deionized water, dried and recrystallized from methanol/water to obtain the final product.

(*E*)-5-phenyl-3-styryl-4,5-dihydro-1-H-pyrazol-1-ethan-1-one (**6a**): The spectral data were in agreement with the literature data [50].

(*E*)-5-(4-fluorophenyl)-3-(4-fluorostyryl)-4,5-dihydro-1-H-pyrazol-1-ethan-1-one (**6b**): The spectral data were in agreement with the literature data [52].

(*E*)-5-(4-bromophenyl)-3-(4-bromostyrene)-4,5-dihydro-1-H-pyrazol-1-ethan-1-one (**6c**): The spectral data were in agreement with the literature data [51].

(*E*)-5-(4-chlorophenyl)-3-(4-chlorostyryl)-4,5-dihydro-1-H-pyrazol-1-ethan-1-one (**6d**): The spectral data were in agreement with the literature data [50].

(*E*)-5-(4-dimethylaminophenyl)-3-(4-dimethylaminostyryl)-4,5-dihydro-1-H-pyrazol-1-ethan-1-one (**6e**): The spectral data were in agreement with the literature data [53].

^1^H-NMR and ^13^C-NMR of the novel synthesized derivatives can be found at the Supplementary Material.

### 3.3. Experimental of Physicochemical Studies

#### Experimental Determination of Lipophilicity by Reverse Phase Thin Layer Chromatography (RP-TLC)—Determination of R_M_ Values

In this experiment, TLC silica gel 60 F_254_ 1.05715.0001 commercial glass plates from Merck were used. The plates were first immersed in a 5% *v*/*v* solution of liquid paraffin in petroleum ether and left for 90 s to become completely impregnated with the stable lipophilic phase. They were then activated by standing for 30 min in an oven at 40 °C and kept there until the time of use. A 70% *v*/*v* MeOH/H_2_O mixture was used as the mobile phase. The mobile phase was saturated with a few drops of liquid paraffin before being used. The spots of the compounds were placed on the plate at a distance of 2 cm from the edges of the plate and also from each other. The development of the spots on the plates was carried out in closed chromatography chambers where the atmosphere is saturated with the mobile phase at room temperature. At the end of development, the solvent-formed front was marked and the plates are dried at 80 °C for about 20–30 min. The appearance of the spots was made under an ultraviolet light lamp. The R_f_ values were the average of 5–6 measurements for each compound and assuming that the compounds will have been rotated to different positions on the plates. The R_M_ values were calculated from the corresponding Rf values according to the equation [67].

R_M_ = log [(1/R_f_) − 1] (1)

### 3.4. Biological In Vitro Tests

For all biological tests, 10 mM stock solutions of the test compounds in DMSO were prepared and then diluted for the determination of the IC_50_ values. Measurements were performed twice and the average of these was used. The standard deviation was less than 10%.

#### 3.4.1. Determination of the Reducing Activity of the Stable Radical DPPH

The tested compounds (0.1 mM final concentration) dissolved in DMSO were incubated with a solution of DPPH in absolute ethanol in absence of light. The absorbance at 20 and 60 min at 517 nm was recorded at room temperature [68,69]. NDGA was used as the reference compound.

#### 3.4.2. Anti-Lipid Peroxidation (AAPH) Activity

The interaction of free radicals with cell membrane lipids induces free radical chain reactions, a process called lipid peroxidation [70]. 930 μL of a phosphate buffer solution (0.05 M, pH = 7.4), 10 μL of the sodium linolate solution (16 mM in a buffer solution of Tris-HCl pH = 9) and 10 μL of the tested compounds (stock solutions 100 μΜ) were mixed and the oxidative procedure started after the addition of 50 μL AAPH at 37 °C under air conditions. The conjugated diene’s absorbance was measured at 234 nm at a thermostable spectrophotometer (37 °C) according to previous publication [71].

#### 3.4.3. Inhibition of Soybean Lipoxygenase

To a quartz cell, add 790 μL of Tris-HCl pH = 9.0 buffer, 200 μL of plant-derived lipoxygenase solution (1:9 × 10^−4^ *w*/*v*, in saline), 10 μL of the test compound solution and 100 μL of sodium linoleate solution (3 mM concentration). The absorbance was measured at 234 nm, where the change in absorbance due to the conversion of linoleic acid to 13-hydroxylinolenic acid was recorded. A solution of 790 μL of Tris-HCl pH = 9.0 buffer, 200 μL of a plant-derived lipoxygenase solution (1:9 × 10^−4^ *w*/*v*, in saline), 10 μL of DMSO and 100 μL of sodium linoleate solution (3 mM concentration) was used as a control. NDGA was used as a reference compound [72].

### 3.5. Molecular Docking and Pharmaco-Similarity Studies

#### 3.5.1. Molecular Docking on Soybean Lipoxygenase

The visualization and preprocessing of the protein (PDB ID: 3PZW) was conducted using UCSF Chimera (version 1.15) [73]. Water molecules and extraneous crystallographic material were removed with Chimera. Modeller (v. 10.3) [74] was applied to add missing residues (Met1-Phe2-Ser3-Ala4-Gly5, Glu21-Val22-Asn23-Pro24-Asp25-Gly26-Ser27-Ala28-Val28-Asp29, Ile117-Ser118-Asn119-Gln120). Hydrogen atoms and charges were added with AmberTools [75,76]. The charge on iron was adjusted to +2.0 applying the 12–6 LJ nonbonded model [77]. Histidines His499, His504 and His690 coordinating iron were set neutral (δ-nitrogen protonated state). TIP3P explicit water model was used. The size of the box has a minimal distance of 12 Å between the solute and the edge of the box. Furthermore, the generation and minimization of ligand 3D coordinates were carried out using Open Babel (version 3.1.1) [78] with the MMFF94 force field [79] ACPYPE [80] was utilized to produce the ligand topologies and parameters using AnteChamber (AmberTools v. 22.10) [81] The energy minimization of the protein was performed using GROMACS (v. 4.6.5) [82]. AutoDock Vina (v. 1.2.3) [83] was used to dock the ligands into the protein. This was achieved by configuring a grid box center x = 1.35 Å, y = 14.3 Å and z = −34.60 Å and size of x = 100 Å, y = 70 Å and z = 70 Å. Moreover, the exhaustiveness parameter was configured with the value of 10 and 20 docking modes were generated as maximum output. UCSF Chimera utilized to analyze the docking results.

#### 3.5.2. Study of Drug-Likeness of Molecules with the Molinspiration Tool

The computational platform Molinspiration (https://www.molinspiration.com/ accessed on 8 April 2024) was used to calculate the physicochemical properties of the compounds synthesized and studied in this research.

## 4. Conclusions

In conclusion, the synthesis of dibenzalacetones was carried out via a double mixed aldol condensation between acetone and suitable substituted benzaldehyde in the presence of sodium hydroxide in ethanol. A total of 32 final dihydro-pyrazoles were synthesized 12 of which were novel in the literature. Compound **1f** was a new one. Compounds **1a**–**f** have been reported in the literature as neuroprotective and antiparasitic agents [42], and were further herein investigated for their antioxidant and lipoxygenase inhibitory activity. The structures of the compounds were identified by melting point measurement and spectroscopic techniques (IR, ^1^H NMR, ^13^C NMR, HRMS, EA). The lipophilicity of the compounds was determined experimentally. Compounds **2e** and **3e** show higher interaction with the DPPH free radical, 45–53% at 60 min. For most of the compounds, it appears that the interaction is time dependent. Lipophilicity does not contribute to the activity. It seems that the bulk of the compounds could prevent their interaction with the free radical. All the synthesized derivatives seem to exhibit very good inhibitory ability against lipid peroxidation. The most active inhibitors of lipid peroxidation are **4a** (98%) and **4b** (97%), whereas compounds **2d** and **2e** were the most potent lipoxygenase inhibitors with IC_50_ values 2.5 μM and 0.35 μM accordingly. Lipophilicity seems to favor lipid peroxidation inhibition but does not influence the lipoxygenase inhibitory activity. Molar refractivity of the substituents governs the lipoxygenase inhibitory activity. The drug likeness of the synthesized derivatives has been studied. Nine compounds have drug likeness properties, while the most potent one violates lipophilicity. Docking studies highlight the interactions with soybean lipoxygenase. Based on the above studies the novel derivatives possess antioxidant and anti-lipoxygenase inhibitory activities while some of them have been reported in the literature as anticancer and anti-inflammatory agents and thus can be considered as possible multitarget agents [42].

## Figures and Tables

**Table 1:** Substituted dibenzalacetones (**1a**–**f**), dihydro-pyrazoles (**2a**–**f**, **3a**–**f**, **4a**–**f**), pyrazole-carboxamides (**5a**–**e**) and dihydro-pyrazol-ethanones (**6a**–**e**). Experimental R_M_ lipophilicity values and theoretical clog *P* lipophilicity values. π_x_ (lipophilic contribution of the X-substituent) and MR_x_ (molar refractivity of the X substituent) [54,55,56,57].

Compd	General Structure	X	Y	R_M_ ± SD	clog *P*	π_x_	MR_x_
**1a**	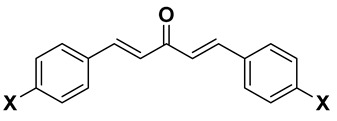	H	-	0.10 ± 0.003	4.66	0	0.103
**1b**		F	-	0.19 ± 0.001	4.94	0.14	0.092
**1c**		Br	-	0.65 ± 0.034	6.38	0.86	0.888
**1d**		Cl	-	0.50 ± 0.042	6.08	0.71	0.603
**1e**		N(CH_3_)_2_	-	−0.14 ± 0.009	4.99	0.18	1.555
**1f**		CF_3_	-	0.57 ± 0.031	6.47	0.88	0.502
**2a**	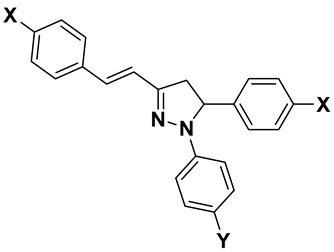	H	H	1.00 ± 0.084	6.90	0	0.103
**2b**		F	H	0.78 ± 0.065	7.19	0.14	0.092
**2c**		Br	H	2.1 ± 0.17	8.63	0.86	0.888
**2d**		Cl	H	1.07 ± 0.067	8.33	0.71	0.603
**2e**		N(CH_3_)_2_	H	0.95 ± 0.092	7.23	0.18	1.555
**2f**		CF_3_	H	1.31 ± 0.031	8.67	0.88	0.502
**3a**		H	Br	1.11 ± 0.068	7.76	0	0.103
**3b**		F	Br	1.03 ± 0.057	8.05	0.14	0.092
**3c**		Br	Br	1.46 ± 0.049	9.49	0.86	0.888
**3d**		Cl	Br	1.39 ± 0.069	9.19	0.71	0.603
**3e**		N(CH_3_)_2_	Br	1.25 ± 0.086	8.09	0.18	1.555
**3f**		CF_3_	Br	1.47 ± 0.044	9.53	0.88	0.502
**4a**		H	CN	−0.31 ± 0.023	6.43	0	0.103
**4b**		F	CN	0.61 ± 0.053	6.62	0.14	0.092
**4c**		Br	CN	0.94 ± 0.069	8.06	0.86	0.888
**4d**		Cl	CN	0.85 ± 0.068	7.76	0.71	0.603
**4e**		N(CH_3_)_2_	CN	0.49 ± 0.029	6.66	0.18	1.555
**4f**		CF_3_	CN	0.8 ± 0.031	8.10	0.88	0.502
**5a**	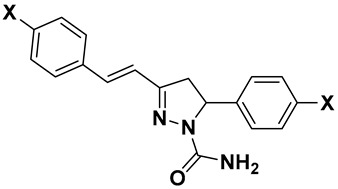	H	-	0.06 ± 0.005	3.24	0	0.103
**5b**		F	-	0.06 ± 0.004	3.52	0.14	0.092
**5c**		Br	-	0.60 ± 0.055	4.96	0.86	0.888
**5d**		Cl	-	0.50 ± 0.043	4.66	0.71	0.603
**5e**		N(CH_3_)_2_	-	0.28 ± 0.019	3.57	0.18	1.555
**6a**	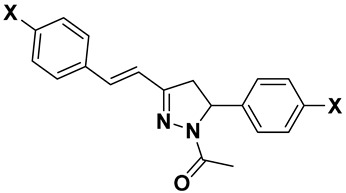	H	-	0.17 ± 0.014	4.11	0	0.103
**6b**		F	-	0.24 ± 0.021	4.40	0.14	0.092
**6c**		Br	-	0.78 ± 0.051	5.84	0.86	0.888
**6d**		Cl	-	0.65 ± 0.048	5.54	0.71	0.603
**6e**		N(CH_3_)_2_	-	0.47 ± 0.036	4.45	0.18	1.555

SD standard deviation of the mean is less than 10%.

**Table 2:** Drug-likeness studies of synthesized compounds.

Compd	miLogP	TPSA	Natoms	MW	nON	nOHNH	Nviolations	Nrotb	Volume
**1a**	4.18	17.07	18	234.30	1	0	0	4	229.27
**1b**	4.51	17.07	20	270.28	1	0	0	4	239.13
**1c**	5.80	17.07	20	392.09	1	0	1	4	265.04
**1d**	5.54	17.07	20	303.19	1	0	1	4	256.34
**1e**	4.39	23.55	24	320.44	3	0	0	6	321.08
**1f**	5.97	17.07	26	370.29	1	0	1	6	291.86
**2a**	6.02	15.6	25	324.43	2	0	1	4	313.11
**2b**	6.34	15.6	27	360.41	2	0	1	4	322.97
**2c**	7.63	15.6	27	482.22	2	0	1	4	348.88
**2d**	7.37	15.6	27	393.32	2	0	1	4	340.18
**2e**	6.22	22.08	31	410.56	4	0	1	6	404.92
**2f**	7.81	15.6	33	460.42	2	0	1	6	375.50
**3a**	6.83	15.6	26	403.32	2	0	1	4	331.00
**3b**	7.15	15.6	28	439.3	2	0	1	4	340.86
**3c**	8.37	15.6	28	561.12	2	0	2	4	366.77
**3d**	8.17	15.6	28	472.21	2	0	1	4	358.07
**3e**	7.03	22.08	32	489.46	4	0	1	6	422.81
**3f**	8.48	15.6	34	539.32	2	0	2	6	393.59
**4a**	5.77	39.39	27	349.44	3	0	1	4	329.97
**4b**	6.10	39.39	29	385.42	3	0	1	4	339.83
**4c**	7.39	39.39	29	507.23	3	0	2	4	365.74
**4d**	7.13	39.39	29	418.33	3	0	1	4	357.04
**4e**	5.97	45.87	33	435.57	5	0	1	6	421.78
**4f**	7.56	39.39	35	485.43	3	0	1	6	392.56
**5a**	3.59	58.70	22	291.35	4	2	0	3	271.97
**5b**	4.95	58.70	24	360.24	4	2	0	3	299.05
**5c**	5.21	58.70	24	449.15	4	2	1	3	307.74
**5d**	3.92	58.70	24	327.33	4	2	0	3	281.84
**5e**	3.80	65.17	28	377.49	6	2	0	5	363.79
**6a**	3.68	32.67	22	290.37	3	0	0	3	277.25
**6b**	5.04	32.67	24	359.26	3	0	1	3	304.32
**6c**	5.30	32.67	24	448.16	3	0	1	3	313.02
**6d**	4.01	32.67	24	326.35	3	0	0	3	287.11
**6e**	3.77	39.15	28	376.50	5	0	0	5	369.06

**Table 3:** Interaction with DPPH at 20 and 60 min (RA%), AAPH-induced linoleic acid peroxidation inhibitory activity (AAPH%), soybean LOX in vitro inhibitory activity (% and IC_50_) of the synthesized compounds.

Compd	RA%, 100 μM, 20 min (±SD)	RA%, 100 μM, 60 min (±SD)	AAPH% Inhibition, 100 μM (±SD)	LOX% Inhibition, 100 μM or IC_50_ (μM) (±SD)
** 1a **	5 ± 0.5	10 ± 0.5	69 ± 0.3	10 μΜ ± 0.4
** 1b **	13 ± 0.6	100 ± 1.1	37 ± 0.1	30 ± 0.1
** 1c **	n.a.	44 ± 1.6	61 ± 0.5	28 ± 0.6
** 1d **	86 ± 4.5	95 ± 4.8	53 ± 0.6	38 ± 0.7
** 1e **	n.a.	100 ± 8.7	50 ± 0.5	24 ± 0.6
** 1f **	13 ± 0.8	26 ± 0.9	32 ± 1.4	24 ± 1.4
** 2a **	n.a.	17 ± 1.2	44 ± 0.7	42 ± 2.4
** 2b **	2 ± 0.1	15 ± 0.6	39 ± 0.3	24 ± 1.9
** 2c **	21 ± 0.6	n.a.	68 ± 0.6	n.a
** 2d **	6 ± 0.4	10 ± 0.9	75 ± 0.1	2.5 μΜ ± 0.2
** 2e **	24 ± 0.9	45 ± 1	78 ± 0.4	0.35 μΜ ± 0.02
** 2f **	5 ± 0.2	n.a.	52 ± 2.5	14 ± 0.8
** 3a **	10 ± 0.3	22 ± 0.9	75 ± 1.4	51 ± 2.2
** 3b **	2 ± 0.1	6 ± 0.6	63 ± 2.6	17 ± 0.8
** 3c **	2 ± 0.1	24 ± 0.9	40 ± 1.8	30 μΜ ± 2.9
** 3d **	6 ± 0.5	11 ± 0.9	52 ± 2.5	11 ± 0.8
** 3e **	30 ± 0.9	53 ± 0.9	80 ± 2.9	20 μΜ ± 0.1
** 3f **	5 ± 0.4	14 ± 1.2	75 ± 2.7	38 ± 2.6
** 4a **	n.a.	n.a.	98 ± 0.4	39 ± 0.4
** 4b **	2 ± 0.1	n.a.	97 ± 0.6	n.a
** 4c **	2 ± 0.1	n.a.	59 ± 0.2	17 ± 1.2
** 4d **	23 ± 0.3	2 ± 0.1	51 ± 0.2	85 μΜ ± 4.1
** 4e **	2 ± 0.1	2 ± 0.2	74 ± 0.9	6 ± 0.5
** 4f **	n.a.	n.a.	12 ± 1.1	22 ± 1.8
** 5a **	n.a.	n.a.	29 ± 2.2	4 ± 0.3
** 5b **	19 ± 0.1	n.a.	8 ± 0.2	39 ± 0.7
** 5c **	n.a.	n.a.	85 ± 0.6	37 ± 0.6
** 5d **	n.a.	34 ± 0.5	33 ± 0.5	80 μΜ ± 3.5
** 5e **	20 ± 0.1	n.a.	61 ± 0.9	19 ± 0.4
** 6a **	11 ± 0.6	n.a.	22 ± 1	24 ± 0.8
** 6b **	n.a.	n.a.	46 ± 0.6	26± 0.3
** 6c **	n.a.	n.a.	71 ± 0.4	1± 0.07
** 6d **	n.a.	8 ± 0.7	67 ± 0.4	42 ± 2.7
** 6e **	7 ± 0.5	12 ± 1	56 ± 0.3	40 μΜ ± 0.6
** NDGA **	87	93	-	93 (0.45 μM)
** Trolox **	-	-	92	-

Standard deviation (±SD) of the mean is less than 10%. For the in vitro assays the mean value is derived from at least six independent experiments.

## Data Availability

Not applicable. The original contributions presented in this study are included in the article. Further inquiries can be directed to the corresponding author(s).

## Supplementary Materials

The following supporting information can be downloaded at: https://www.mdpi.com/article/10.3390/molecules30102224/s1, Table S1. Substituted dibenzalacetones (**1a**–**f**), dihydro-pyrazoles (**2a**–**f**, **3a**–**f**, **4a**–**f**), pyrazole-carboxamides (**5a**–**e**) and dihydro-pyrazol-ethanones (**6a**–**e**); ^1^H-NMR and ^13^C-NMR of the novel synthesized derivatives (Refs. [44,45,46,47,48,49,50,51,52,53,67]).
