# Supplementary material for: Microwave Assisted Synthesis of Antioxidant Dihydro-Pyrazole Hybrids as Possible Lipoxygenase Inhibitors
Source: Molecules. 2025 May 20;30(10):2224. doi: 10.3390/molecules30102224 (PMC12113707; doi:10.3390/molecules30102224)

Supporting information about the general stuctures, substitution, yields and references of the novel derivatives

Table S1. Substituted dibenzalacetones (**1a-f**), dihydro-pyrazoles (**2a-f, 3a-f, 4a-f**), pyrazole-carboxamides **(5a-e)** and dihydro-pyrazol-ethanones **(6a-e).**

compd	General Structure	X	Y	A%	Reference
**1a**	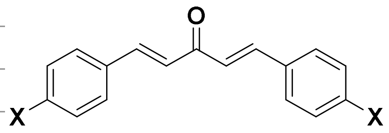	H	-	68	[44]
**1b**		F	-	61	[45]
**1c**		Br	-	80	[46]
**1d**		Cl	-	78	[46]
**1e**		$N(CH_3)_2$	-	90	[47]
**1f**		CF_3	-	30	-
**2a**	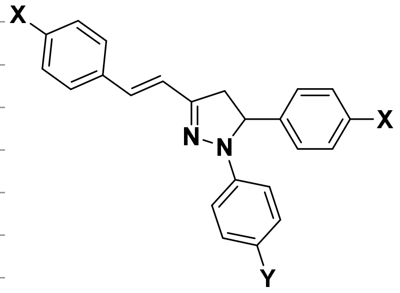	H	H	50	[43]
**2b**		F	H	62	[48]
**2c**		Br	H	58	[49]
**2d**		Cl	H	71	[49]
**2e**		$N(CH_3)_2$	H	65	[43]
**2f**		CF_3	H	50	-
**3a**		H	Br	82	[67]
**3b**		F	Br	80	-
**3c**		Br	Br	52	-
**3d**		Cl	Br	68	-
**3e**		$N(CH_3)_2$	Br	52	-
**3f**		CF_3	Br	52	-
**4a**		H	CN	57	[67]
**4b**		F	CN	73	-
**4c**		Br	CN	78	-
**4d**		Cl	CN	75	-
**4e**		$N(CH_3)_2$	CN	55	-
**4f**		CF_3	CN	67	-
**5a**	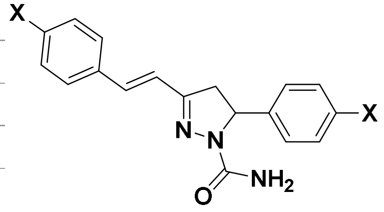	H	-	8	[50]
**5b**		F	-	9	-
**5c**		Br	-	15	-
**5d**		Cl	-	51	[41]
**5e**		$N(CH_3)_2$	-	27	[53]
**6a**	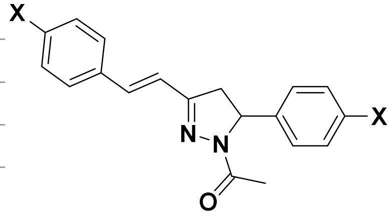	H	-	42	[50]
**6b**		F	-	13	[52]
**6c**		Br	-	38	[51]
**6d**		Cl	-	48	[50]
**6e**		$N(CH_3)_2$	-	23	[53]

Supporting information about the ^{1}H-NMR and ^{13}C-NMR spectra of synthesized derivatives

SP10 corresponds to compound **1f**

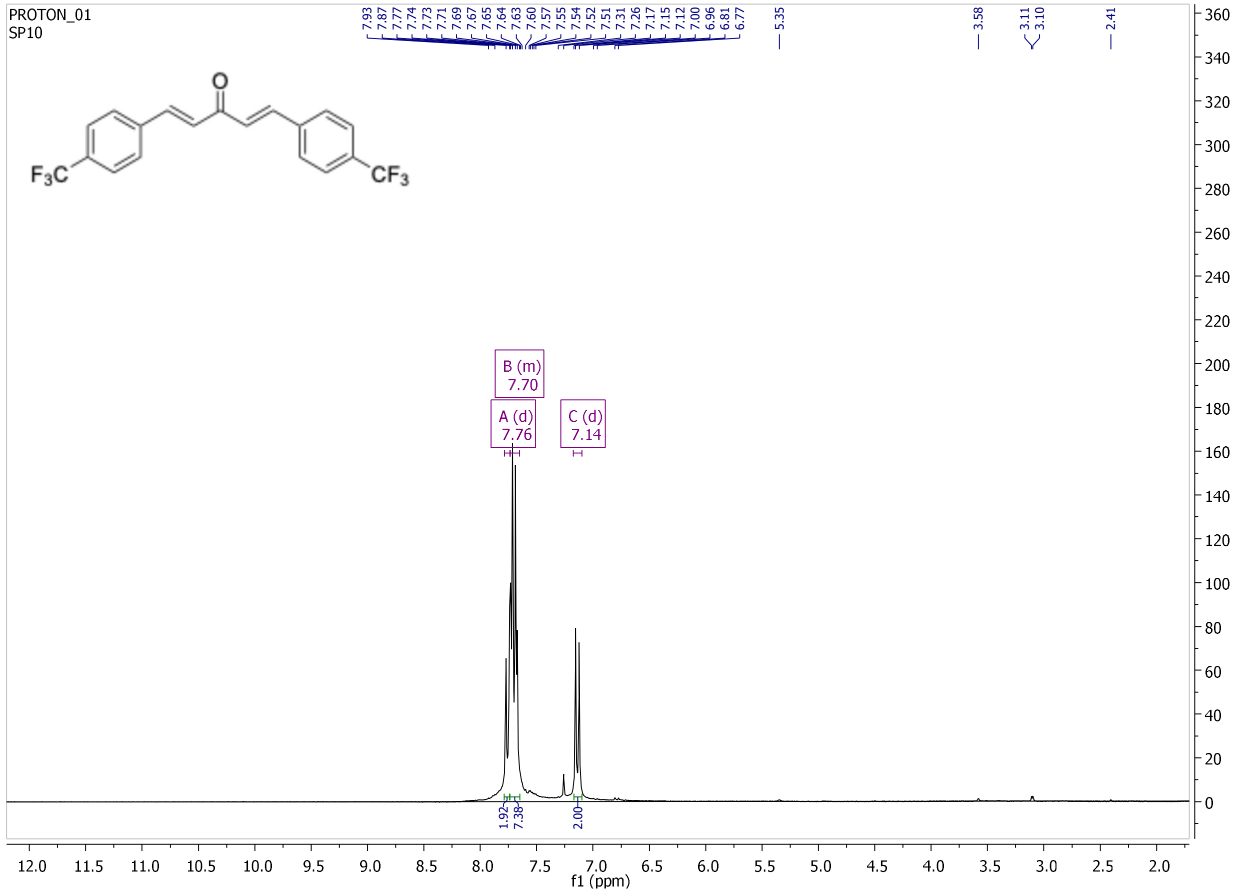

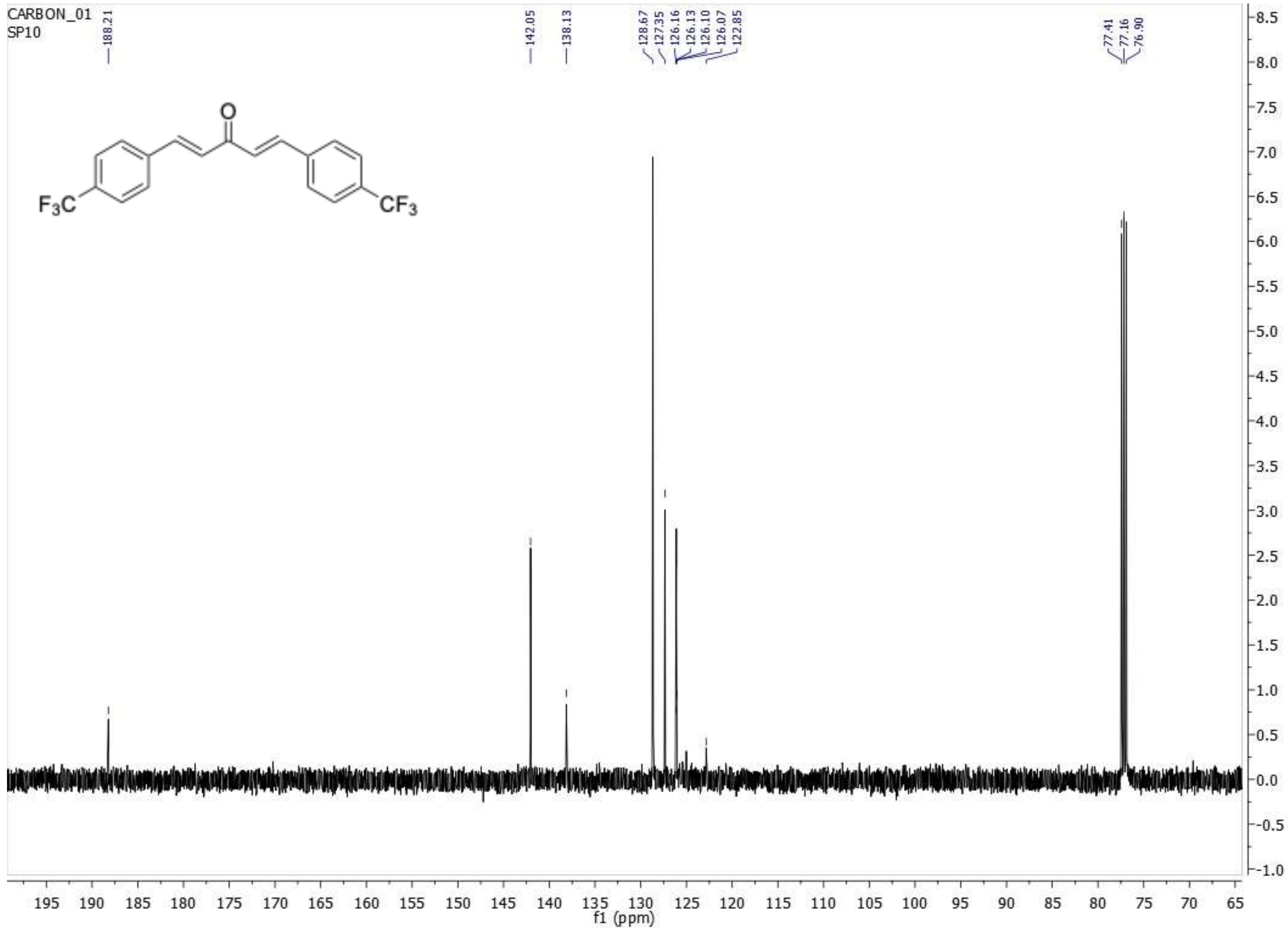
CARBON_01
SP10
188.21
142.05
138.13
128.67
127.35
126.16
126.13
126.10
126.07
122.85
77.41
77.16
76.90
O
F3C
CF3
f1 (ppm)

SX13 corresponds to compound **2f**

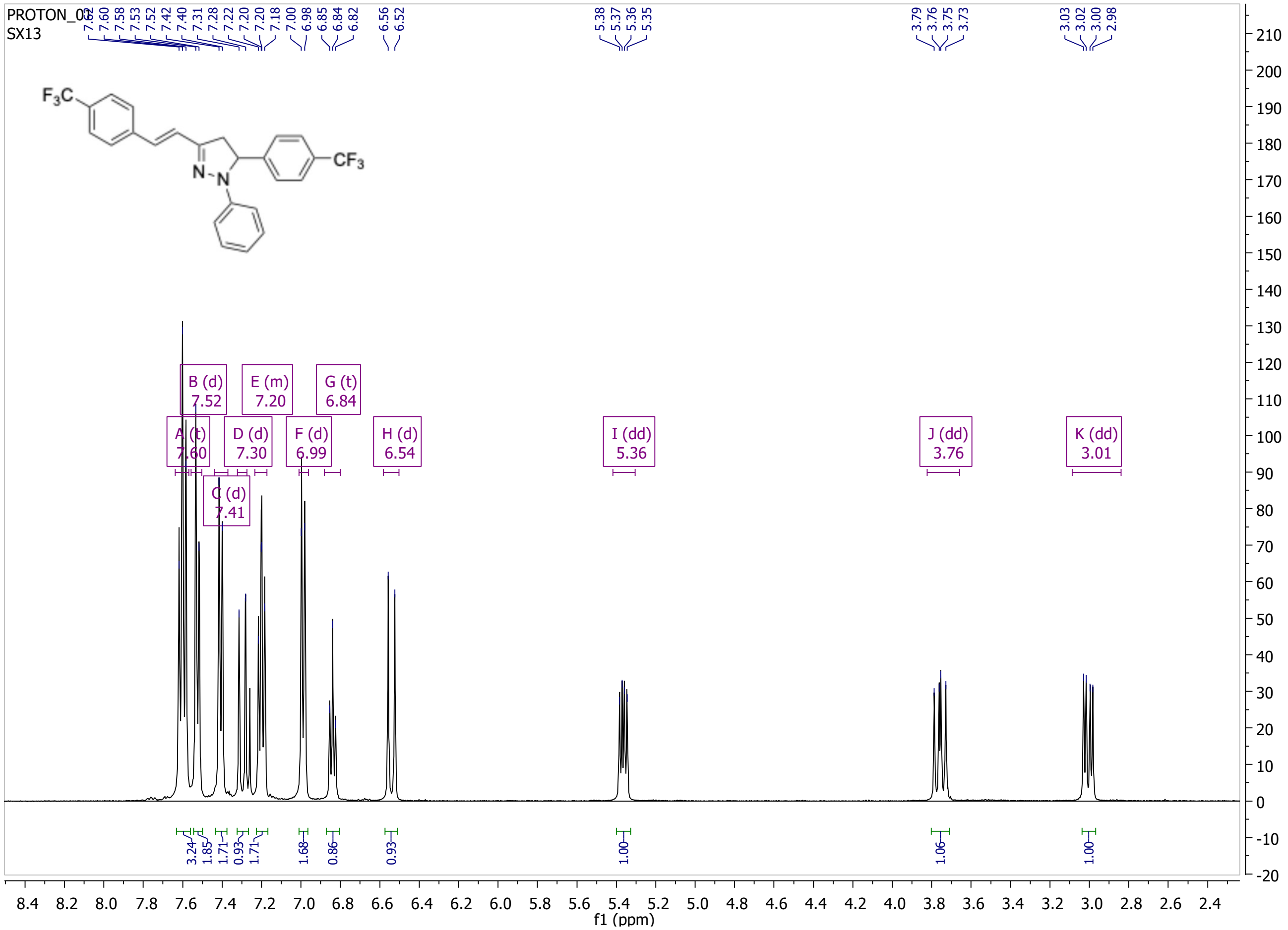

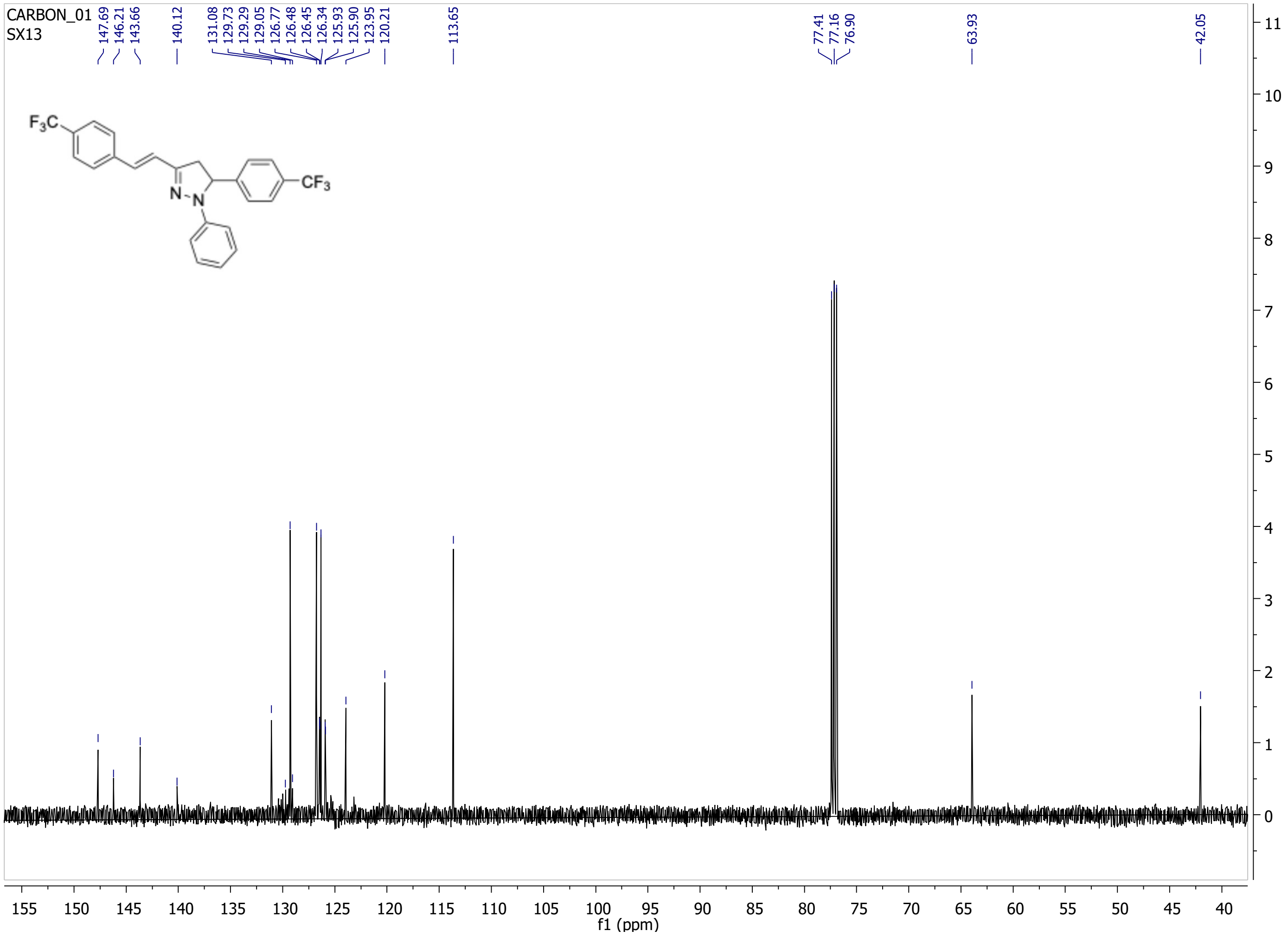
CARBON_01
SX13
147.69
146.21
143.66
140.12
131.08
129.73
129.29
129.05
126.77
126.48
126.45
126.34
125.93
125.90
123.95
120.21
113.65
77.41
77.16
76.90
63.93
42.05
F3C
N
N
CF3
f1 (ppm)

SX4 corresponds to compound **3d**

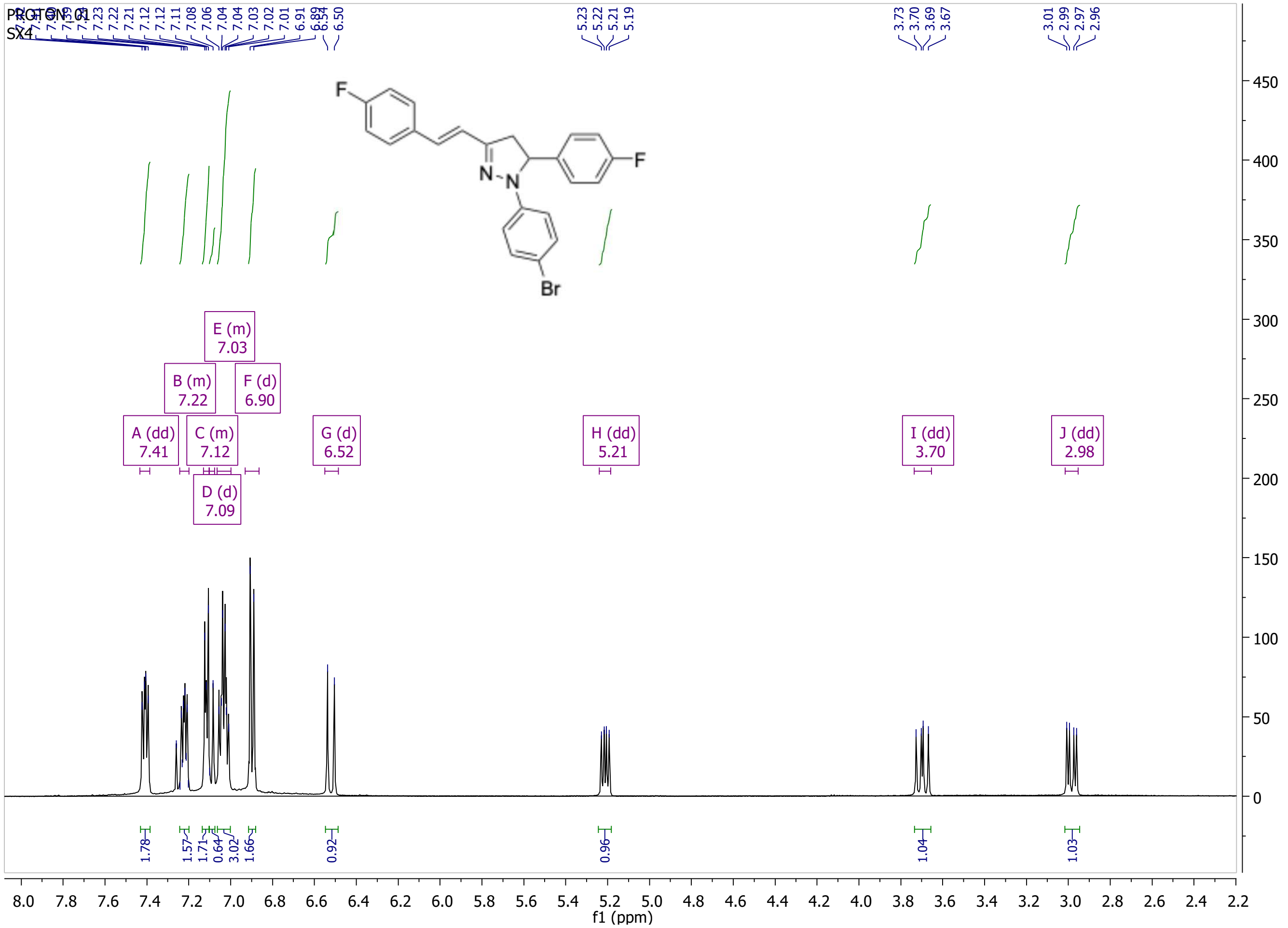

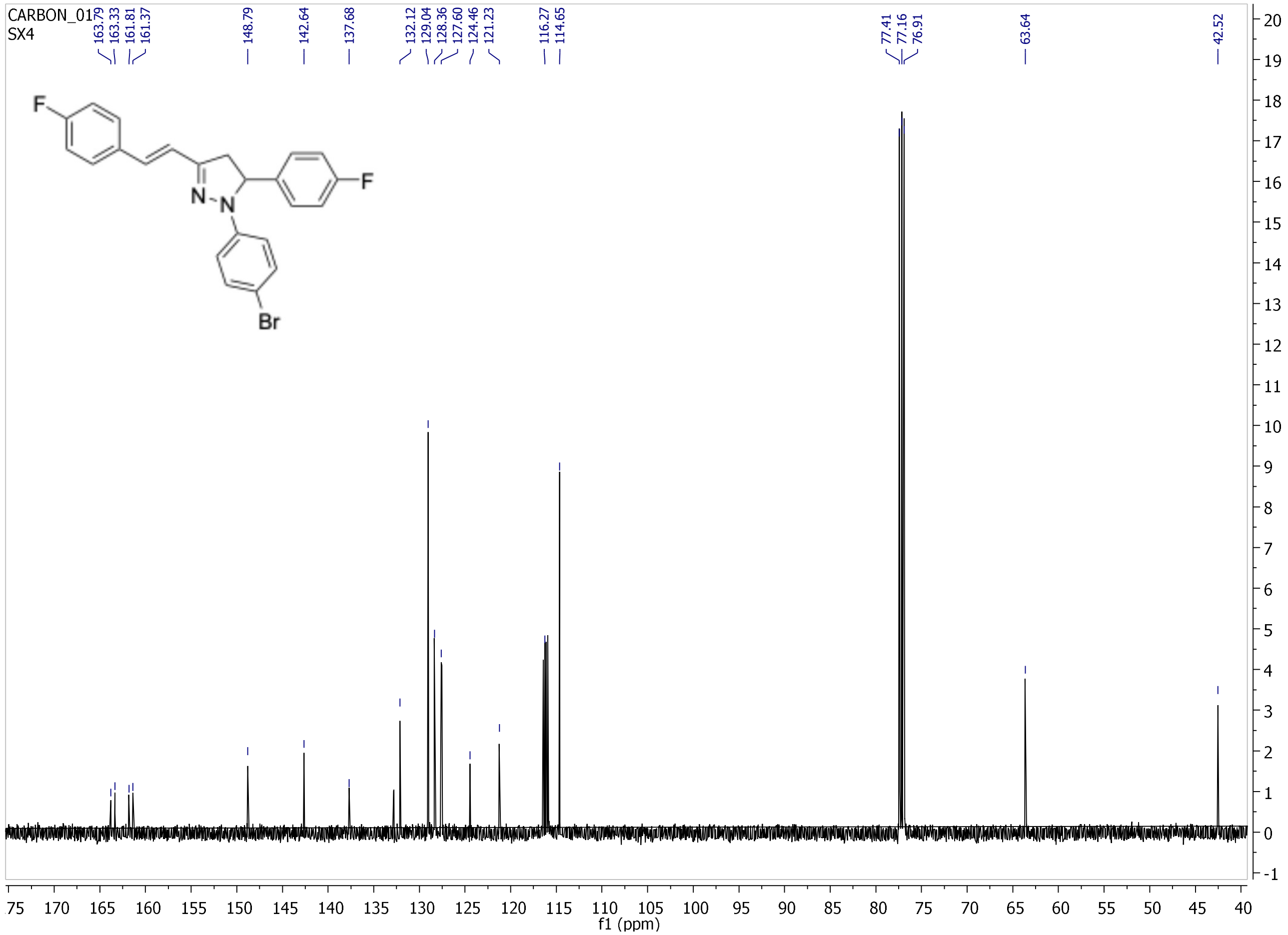
CARBON_01
SX4
163.79
163.33
161.81
161.37
148.79
142.64
137.68
132.12
129.04
128.36
127.60
124.46
121.23
116.27
114.65
77.41
77.16
76.91
63.64
42.52
F
F
N
N
Br
f1 (ppm)

SX10 corresponds to compound **3c**

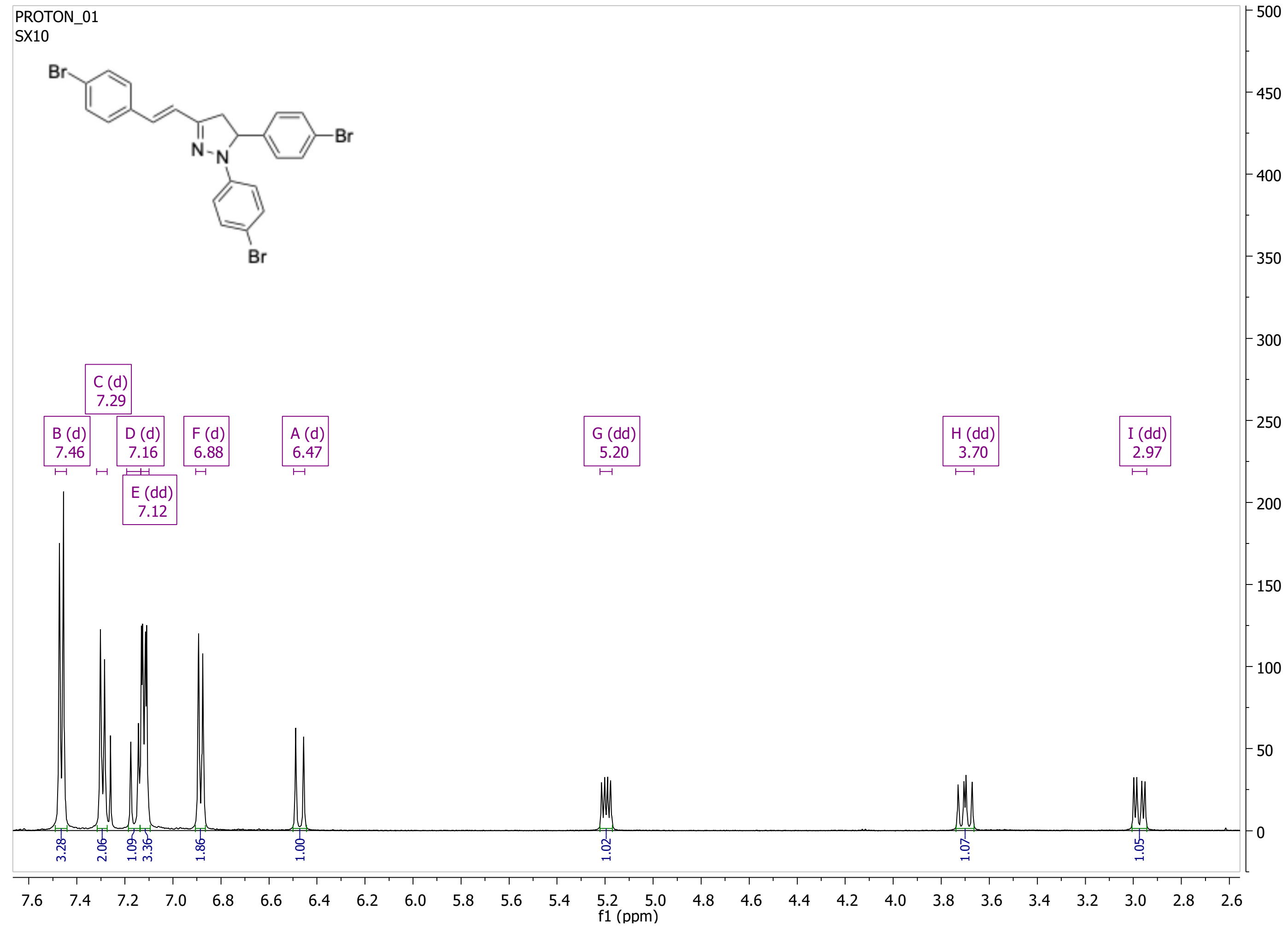

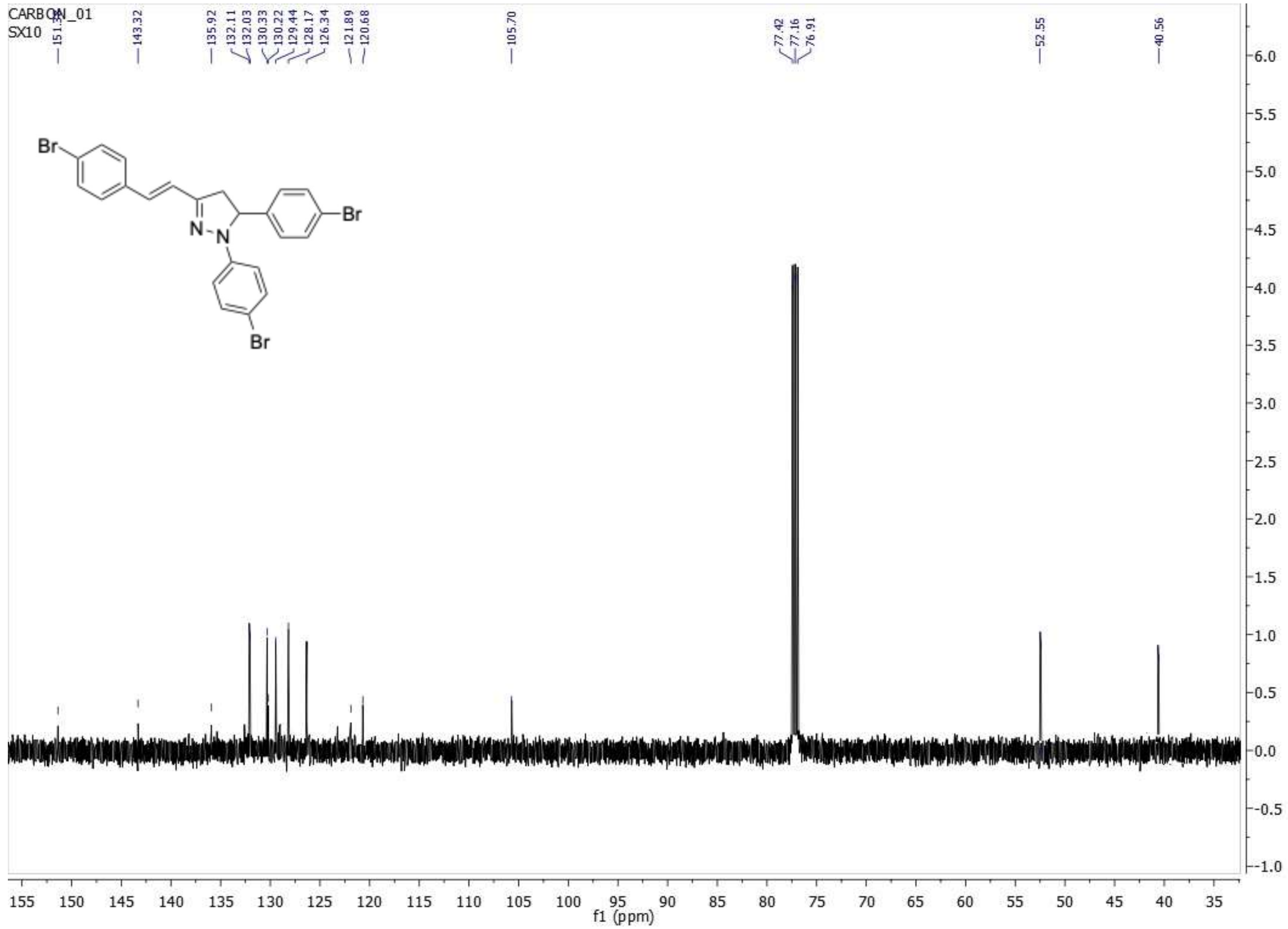
CARBON_01
SX10
151.38
143.32
135.92
132.11
132.03
130.33
130.22
129.44
128.17
126.34
121.89
120.68
105.70
77.42
77.16
76.91
52.55
40.56
Br
Br
N
N
Br
f1 (ppm)

SX24 corresponds to compound **3d**

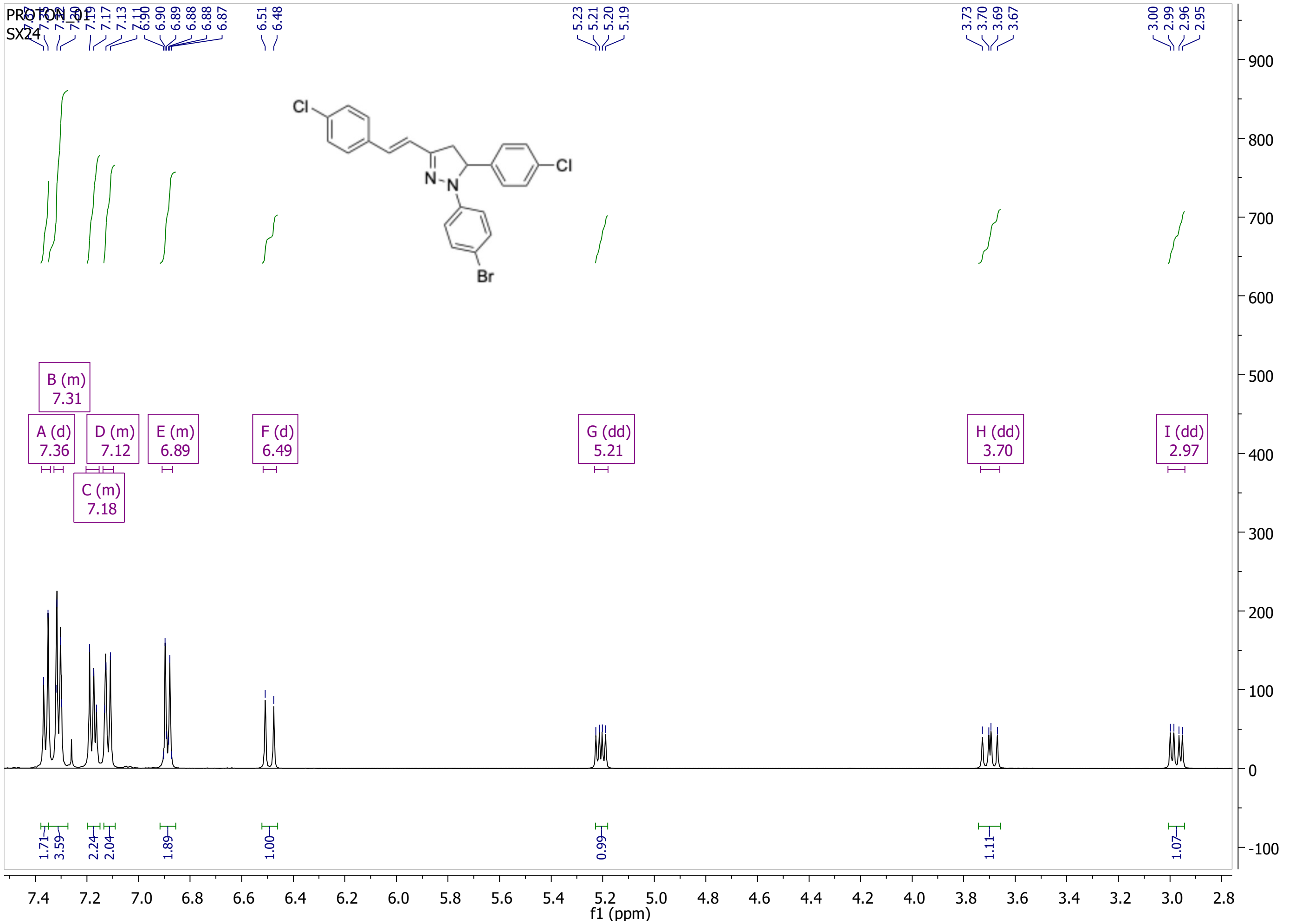

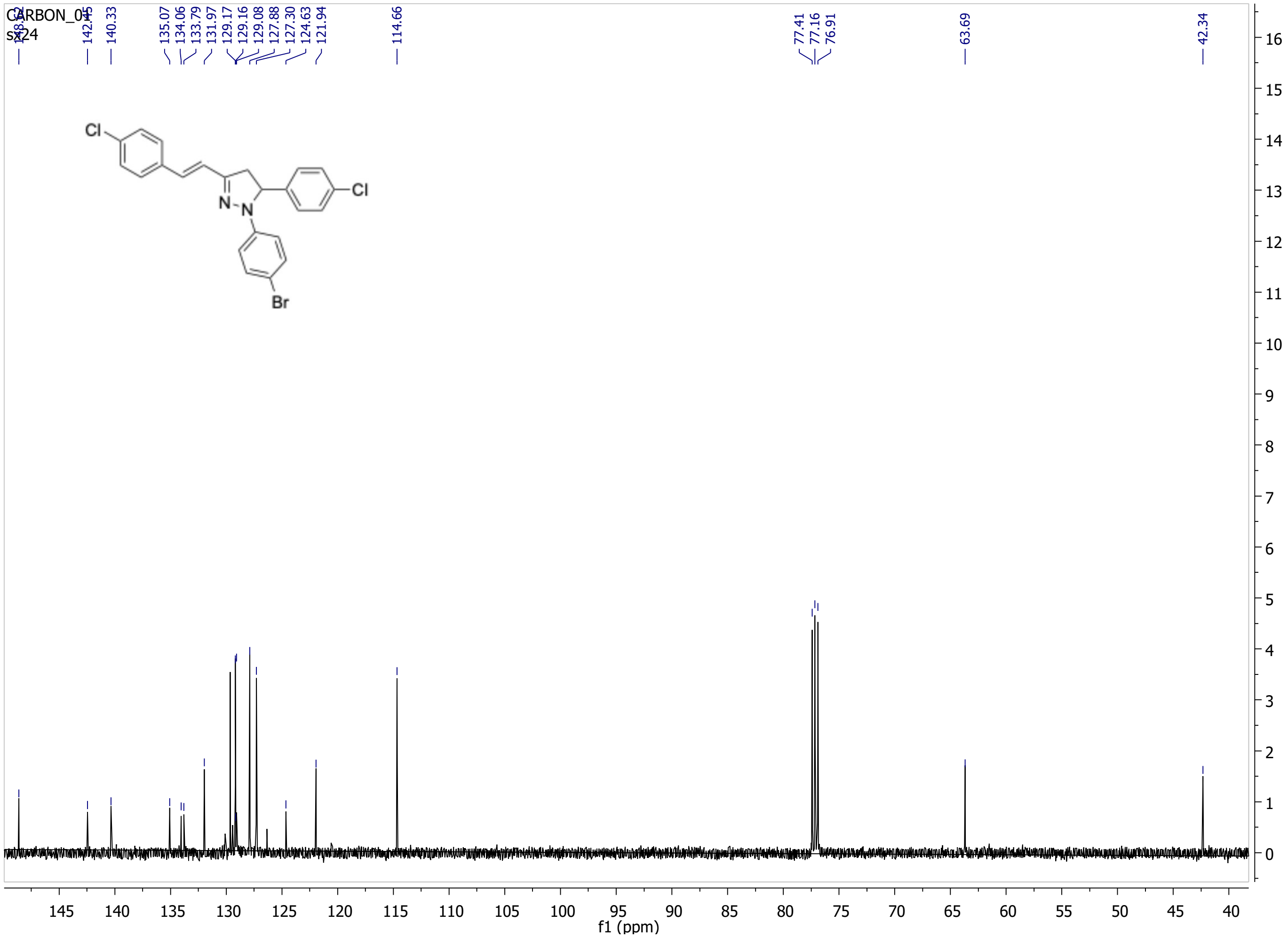
CARBON_01
sx24
140.33
135.07
134.06
133.79
131.97
129.17
129.16
129.08
127.88
127.30
124.63
121.94
114.66
77.41
77.16
76.91
63.69
42.34
Cl
N
N
Cl
Br
f1 (ppm)

SX6 corresponds to compound **3e**

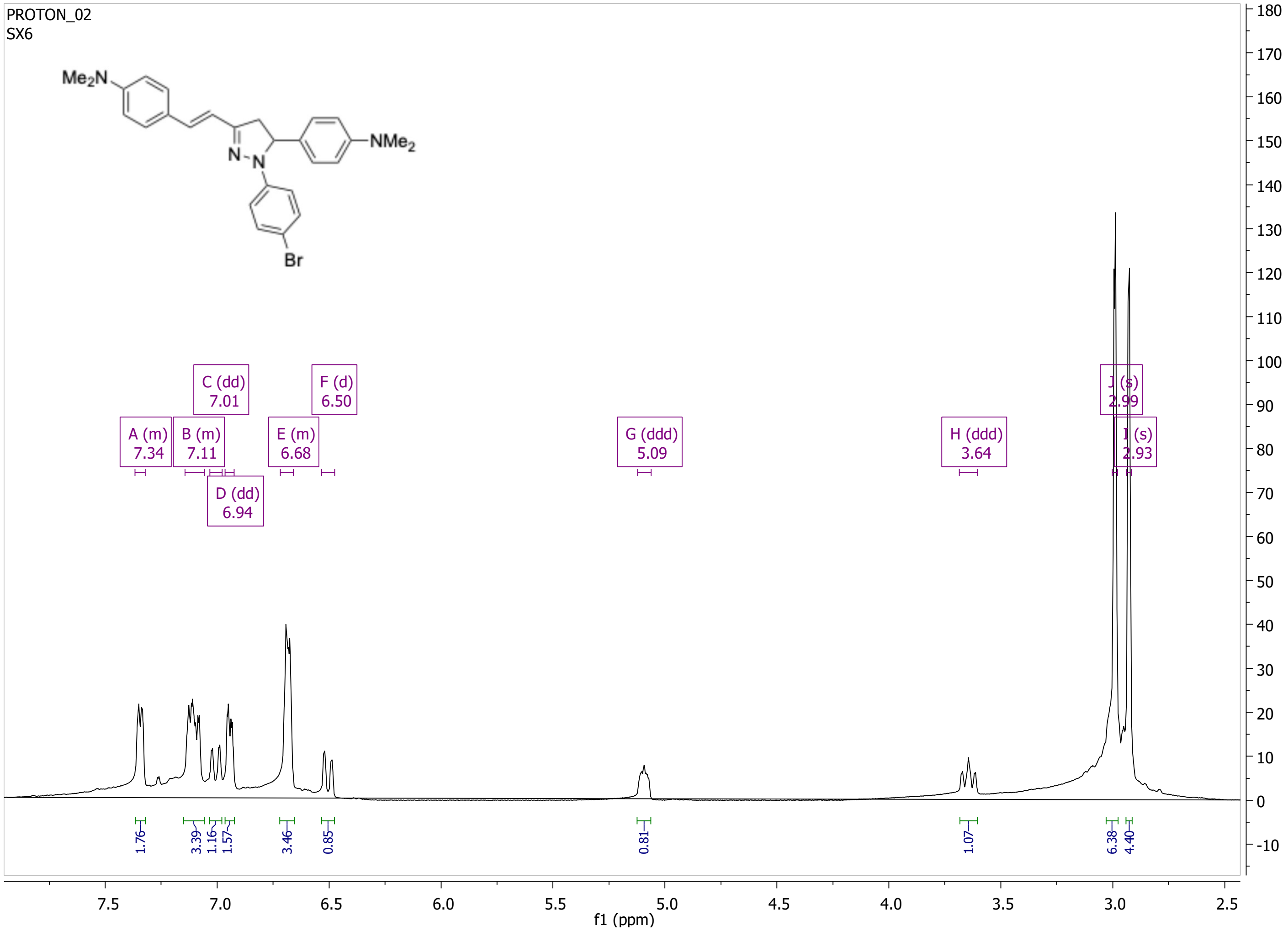

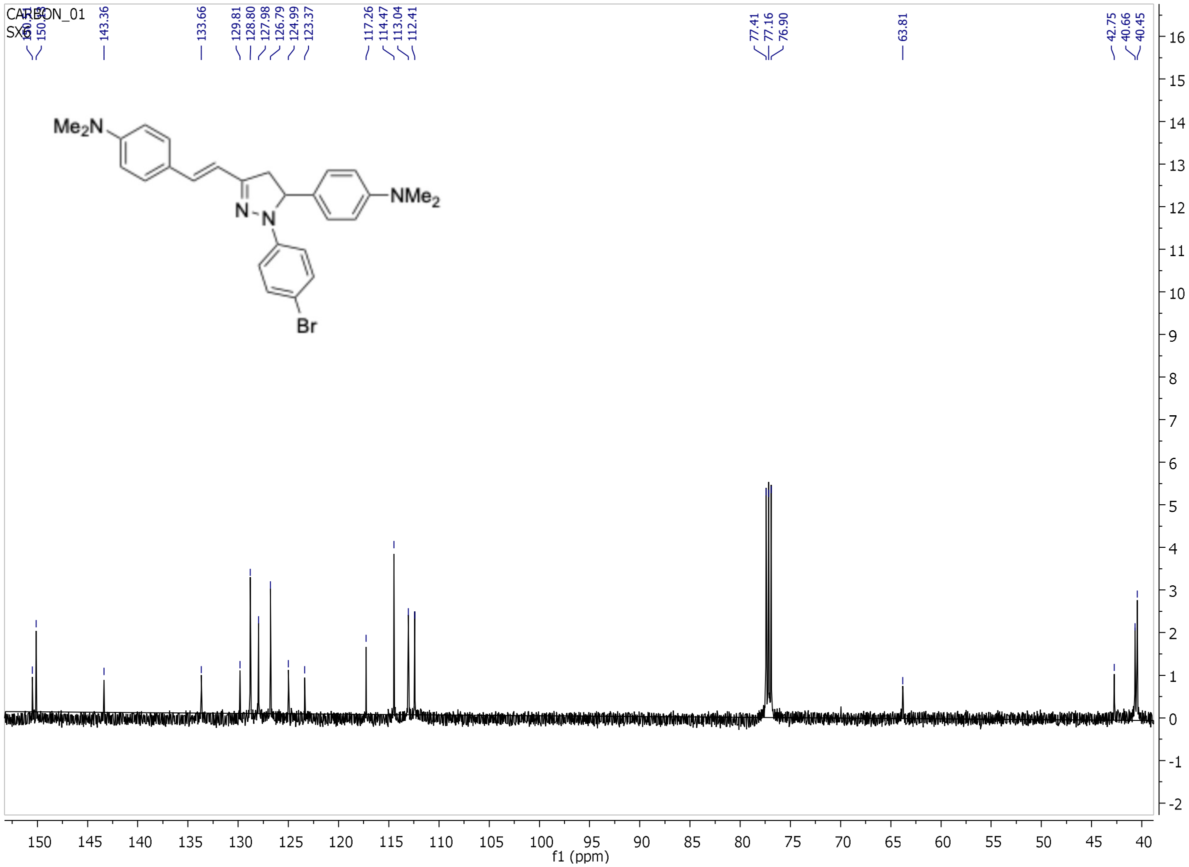
CARBON_01
SX6
143.36
133.66
129.81
128.80
127.98
126.79
124.99
123.37
117.26
114.47
113.04
112.41
77.41
77.16
76.90
63.81
42.75
40.66
40.45
Me2N
NMe2
N
N
Br
f1 (ppm)

SX14 corresponds to compound **3f**

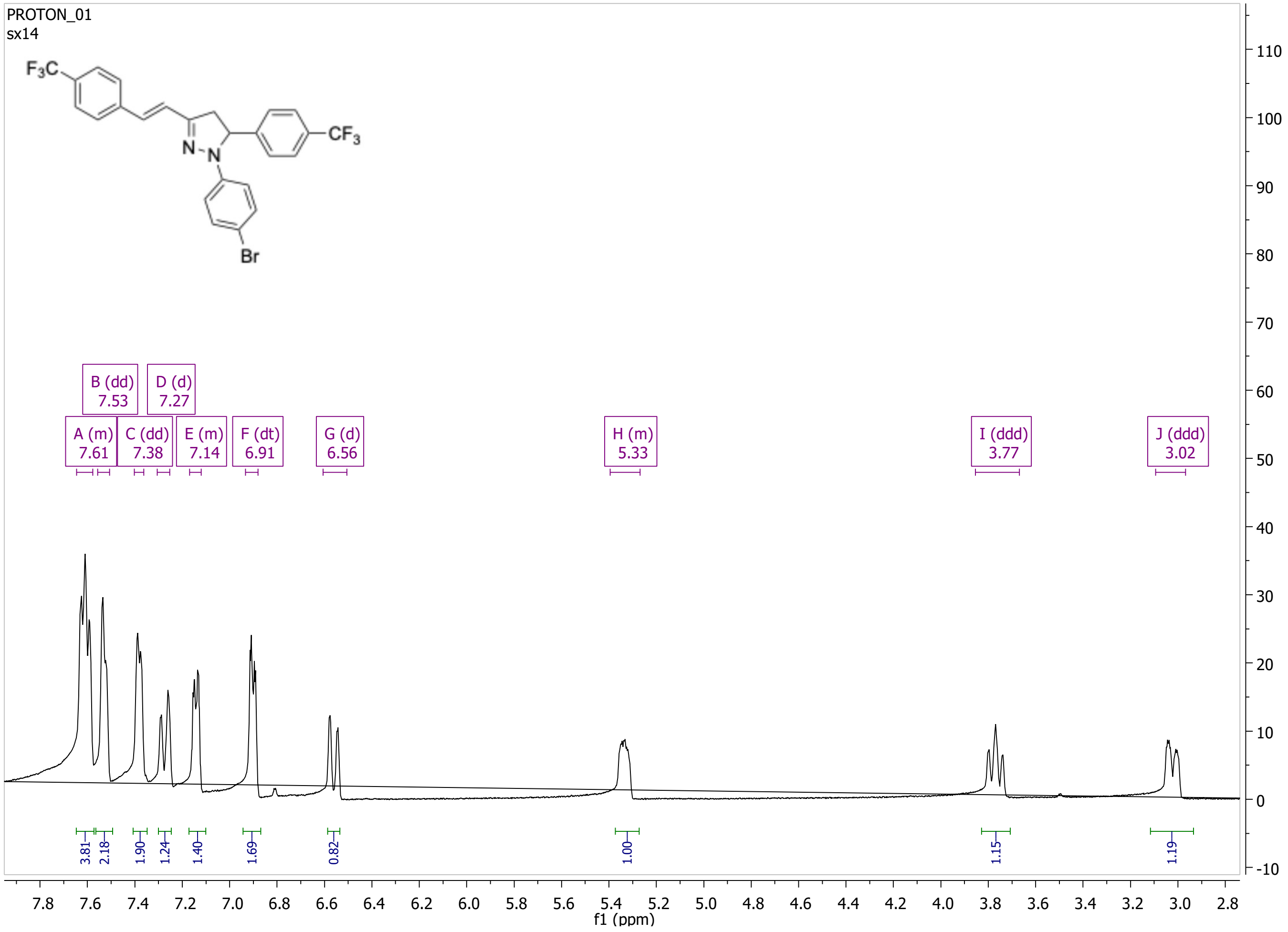

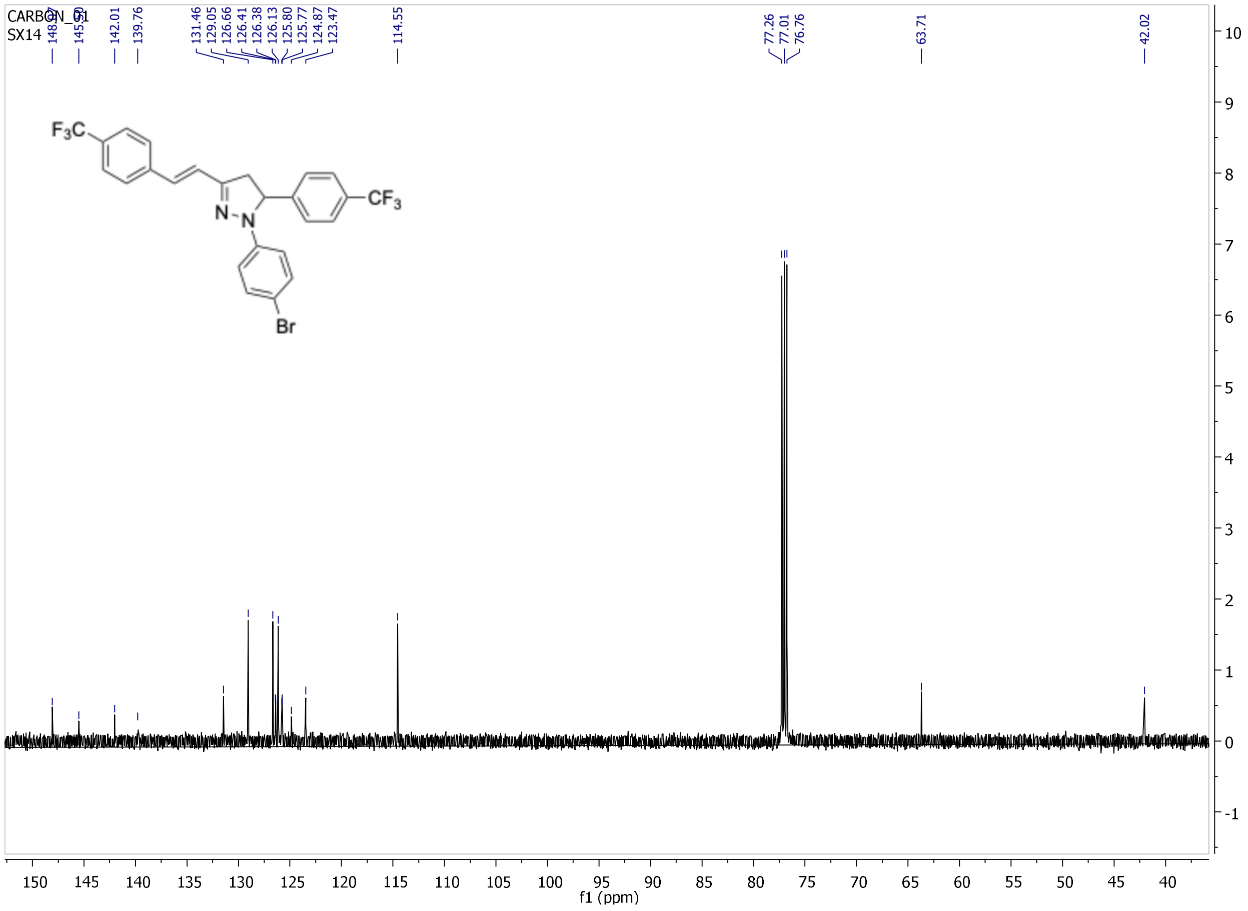

CARBON_01
SX14
142.01
139.76
131.46
129.05
126.66
126.41
126.38
126.13
125.80
125.77
124.87
123.47
114.55
77.26
77.01
76.76
63.71
42.02
F3C
CF3
N
N
Br
f1 (ppm)

SX21 corresponds to compound **4b**

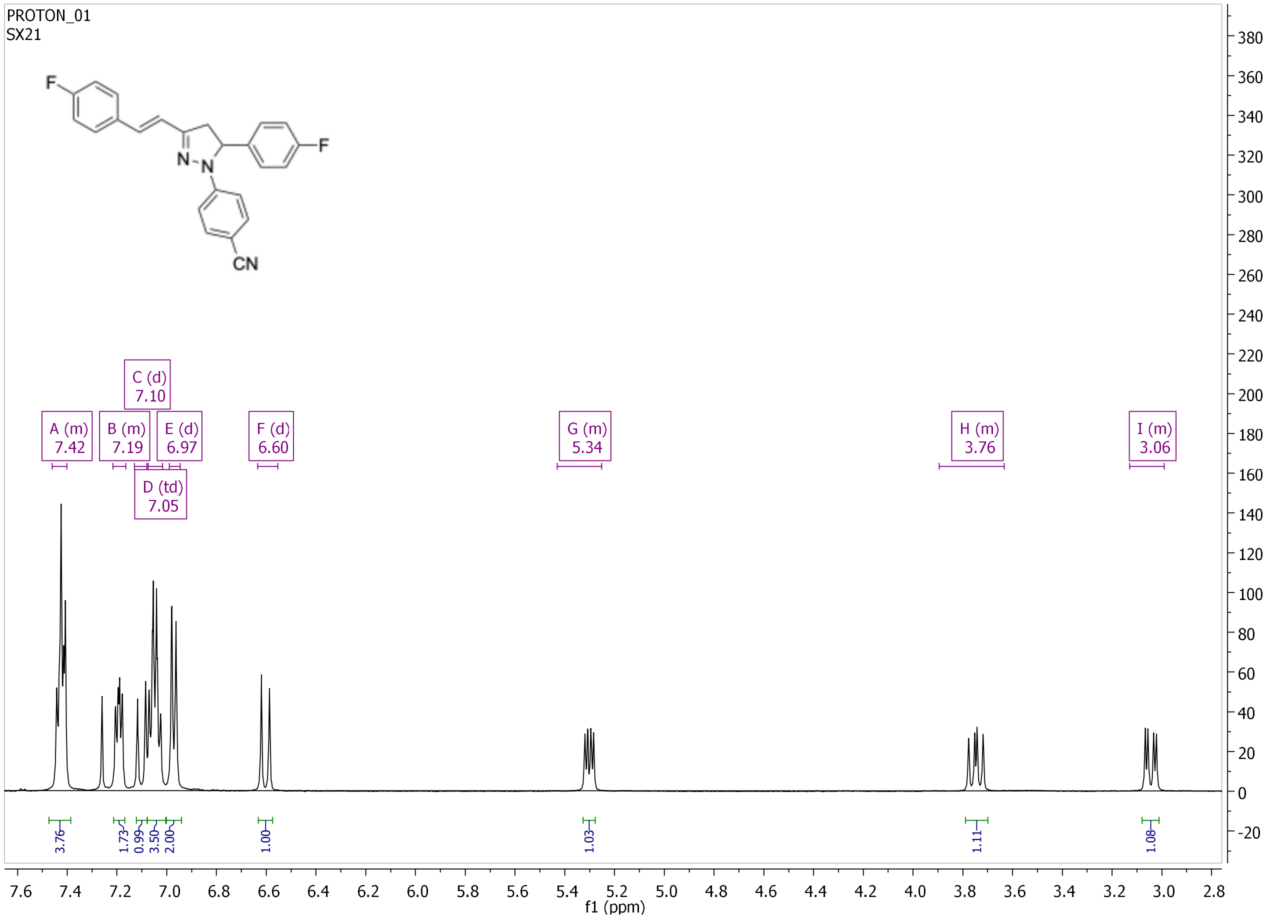

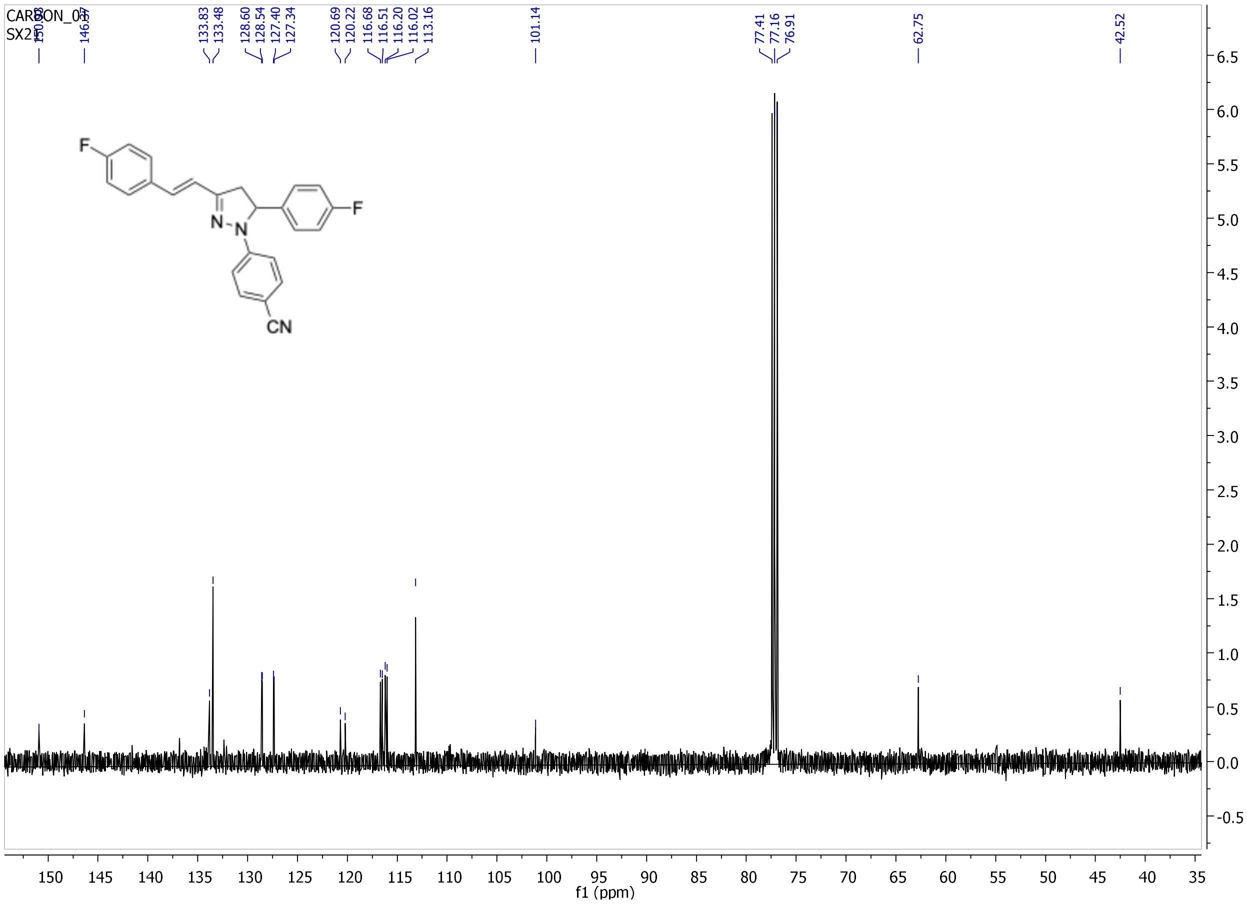
CARBON_01
SX21
150.93
146.37
133.83
133.48
128.60
128.54
127.40
127.34
120.69
120.22
116.68
116.51
116.20
116.02
113.16
101.14
77.41
77.16
76.91
62.75
42.52
F
F
N
N
CN
6.5
6.0
5.5
5.0
4.5
4.0
3.5
3.0
2.5
2.0
1.5
1.0
0.5
0.0
-0.5
150 145 140 135 130 125 120 115 110 105 100 95 90 85 80 75 70 65 60 55 50 45 40 35
f1 (ppm)

SX29 corresponds to compound **4c**

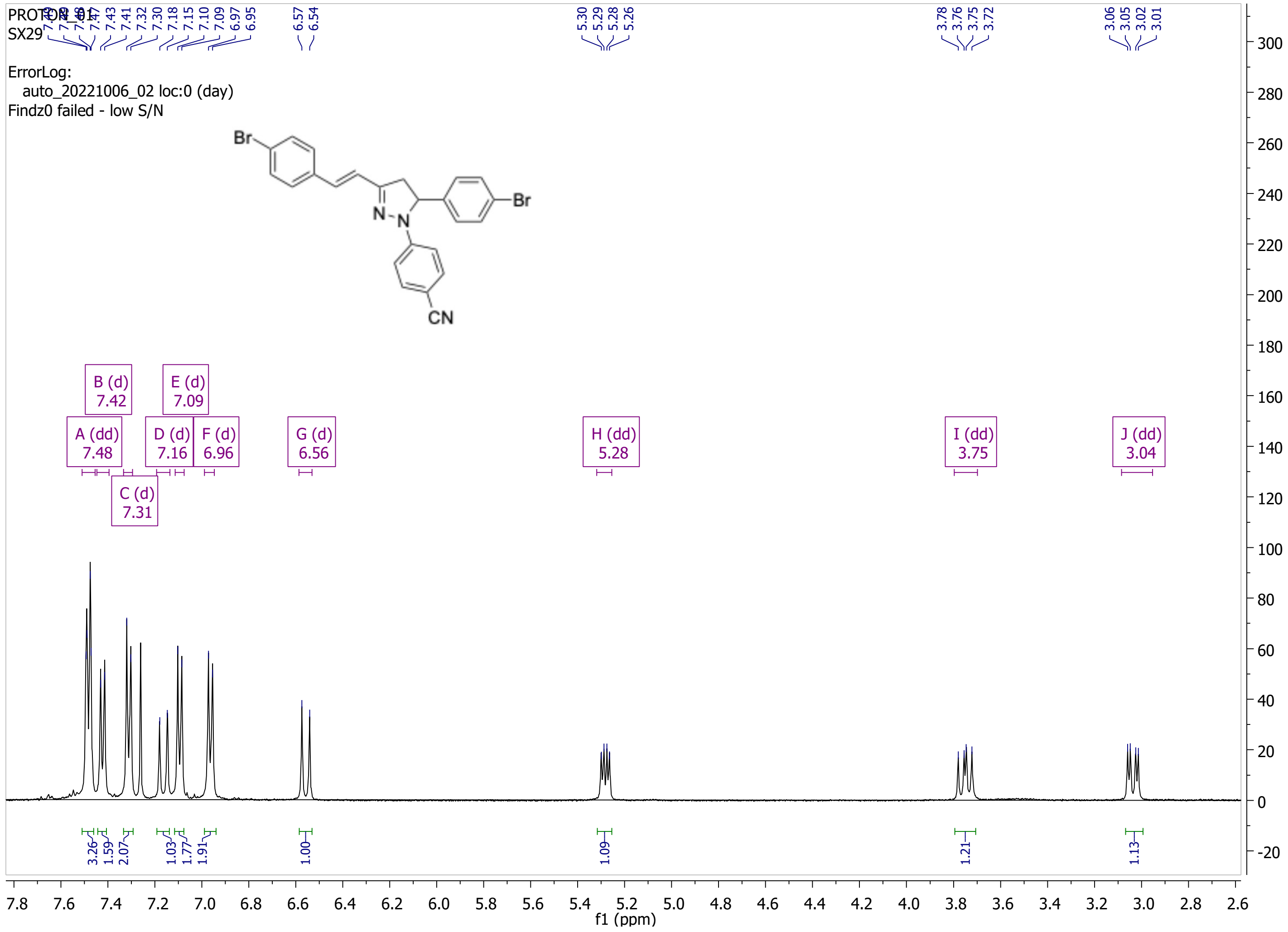

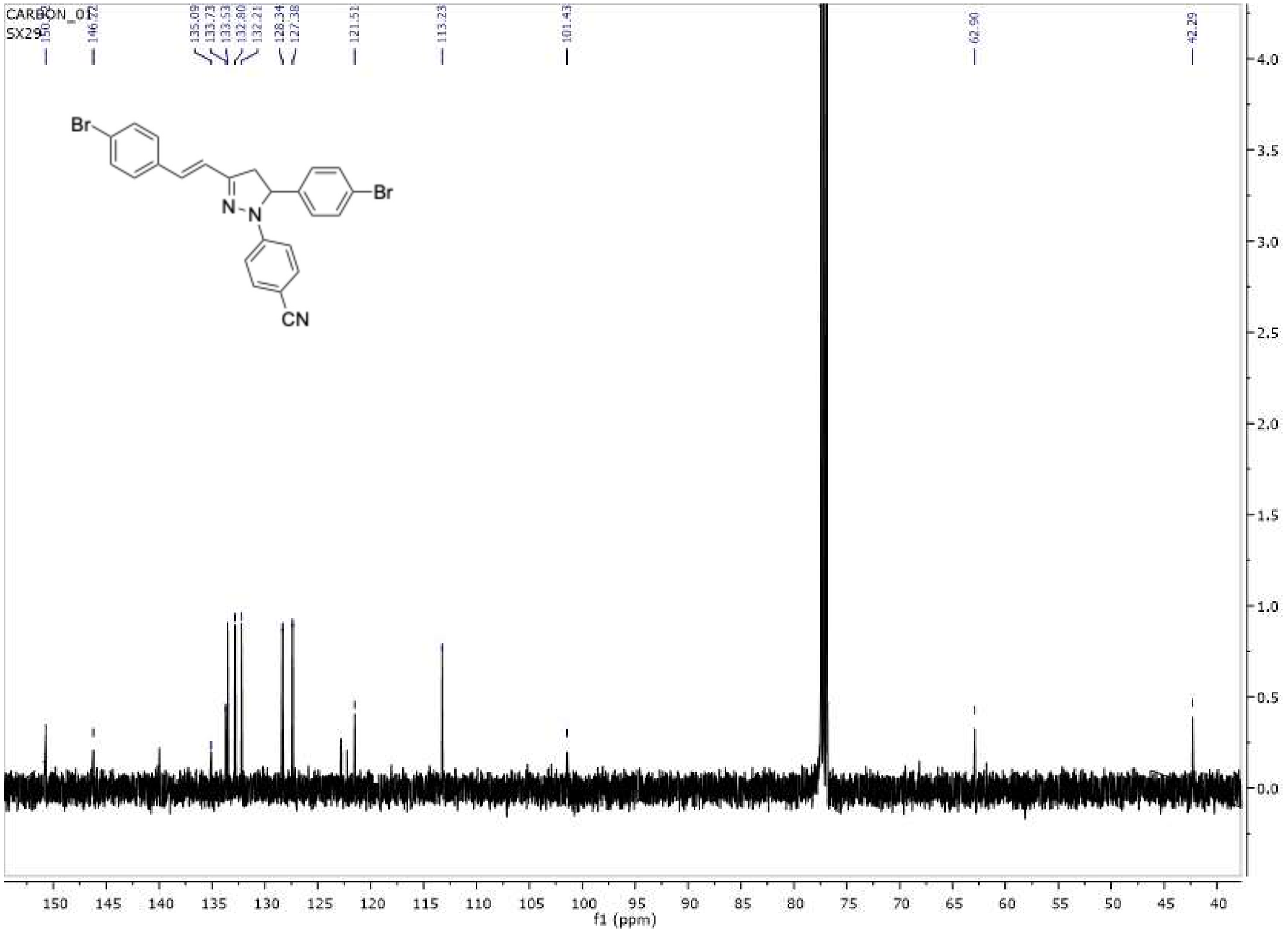

CARBON_01
SX29
135.09
133.73
133.53
132.80
132.21
128.34
127.38
121.51
113.23
101.43
62.90
42.29
Br
Br
N
N
CN
f1 (ppm)

SX28 corresponds to compound **4d**

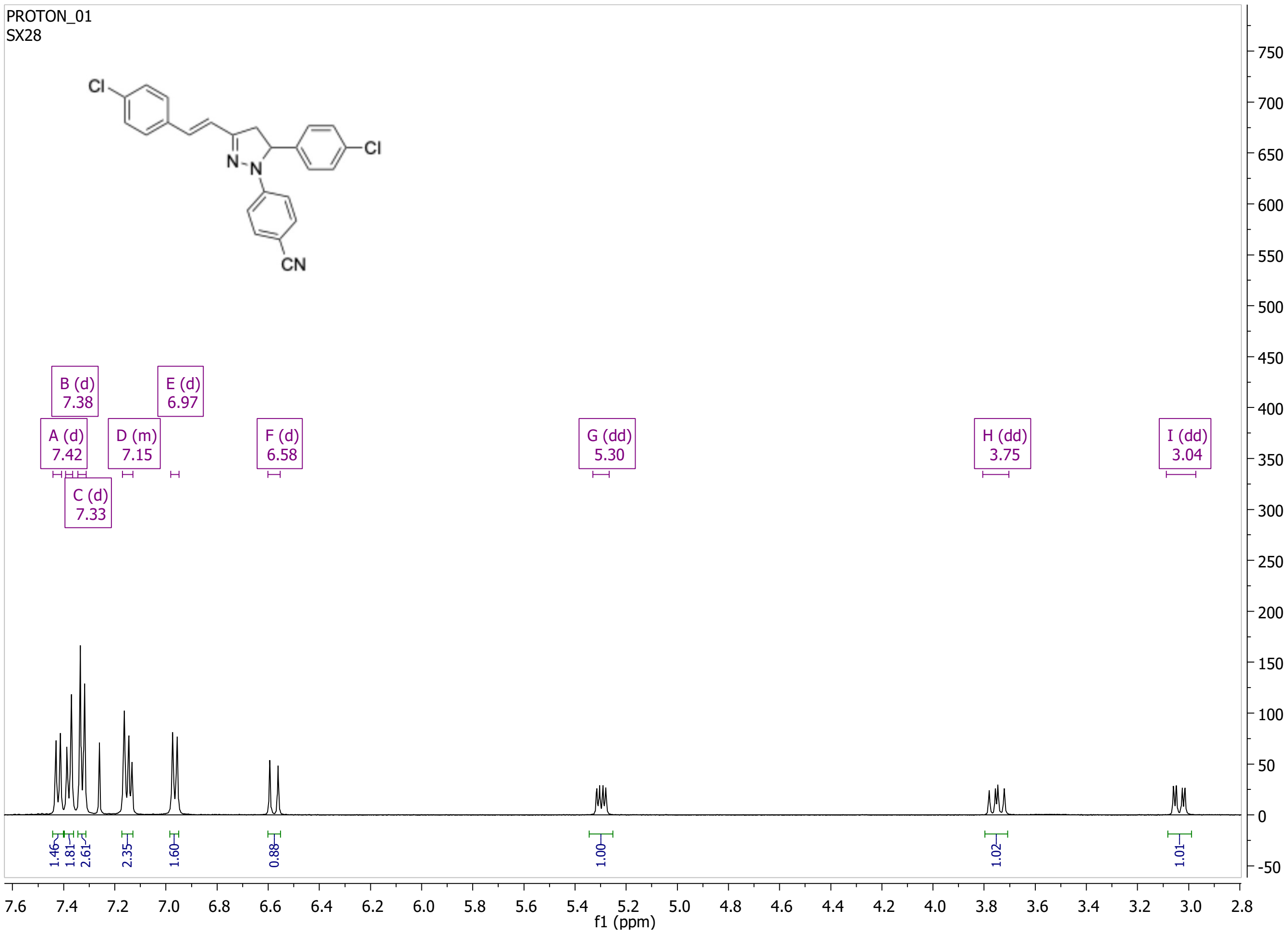

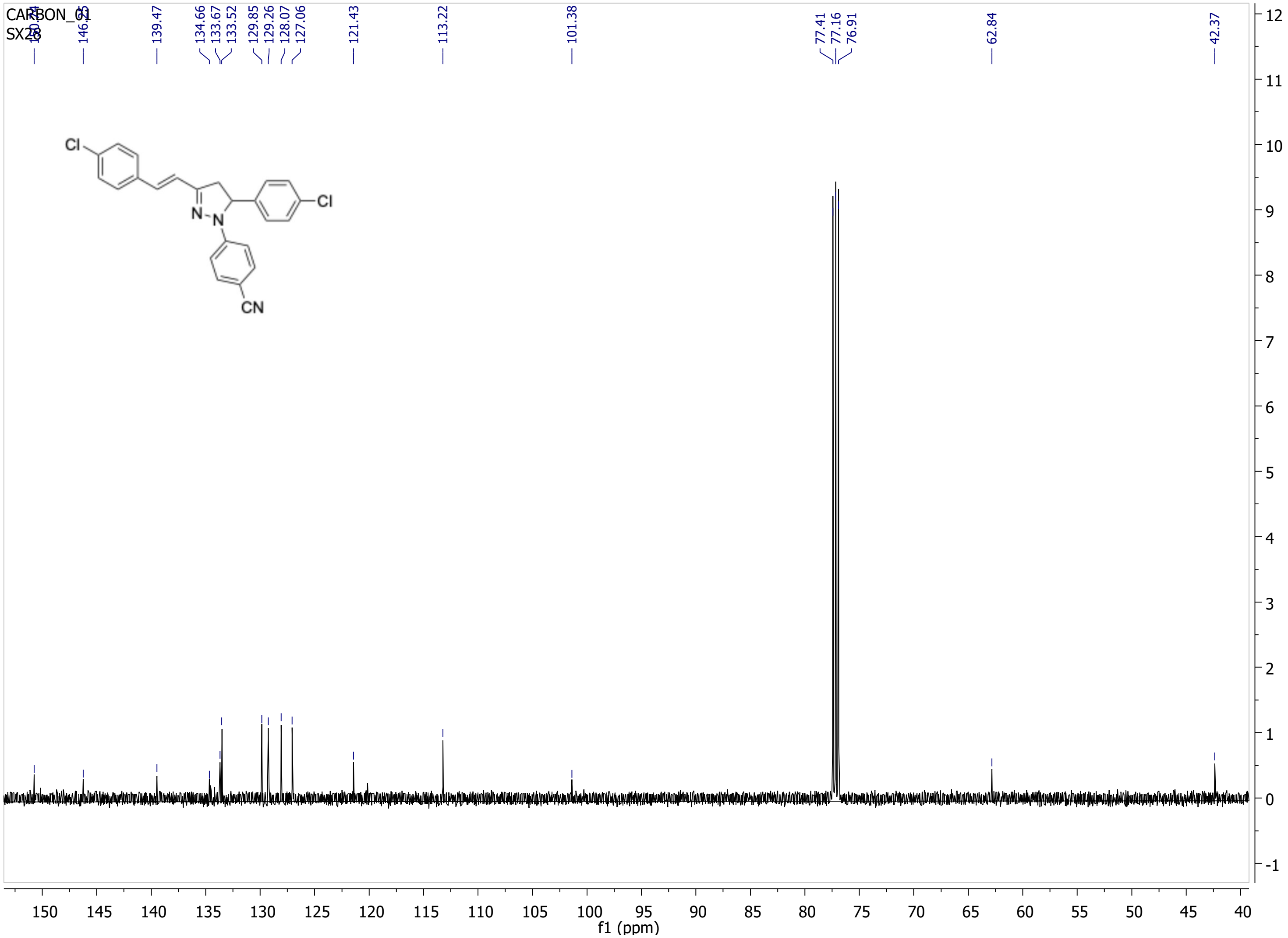
CARBON_01
SX28
150.74
146.25
139.47
134.66
133.67
133.52
129.85
129.26
128.07
127.06
121.43
113.22
101.38
77.41
77.16
76.91
62.84
42.37
Cl
Cl
N
N
CN
f1 (ppm)

SX31 corresponds to compound **4e**

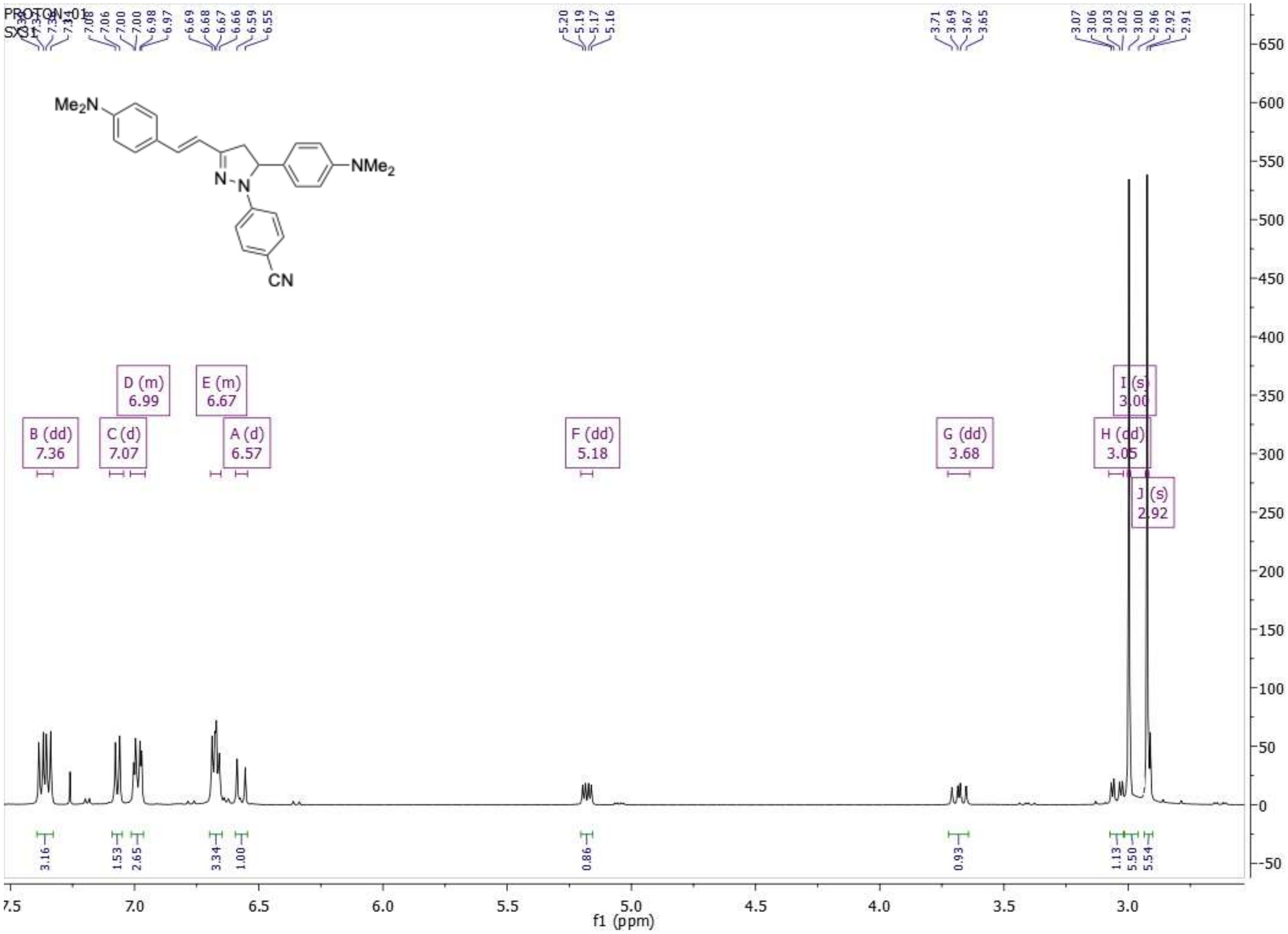

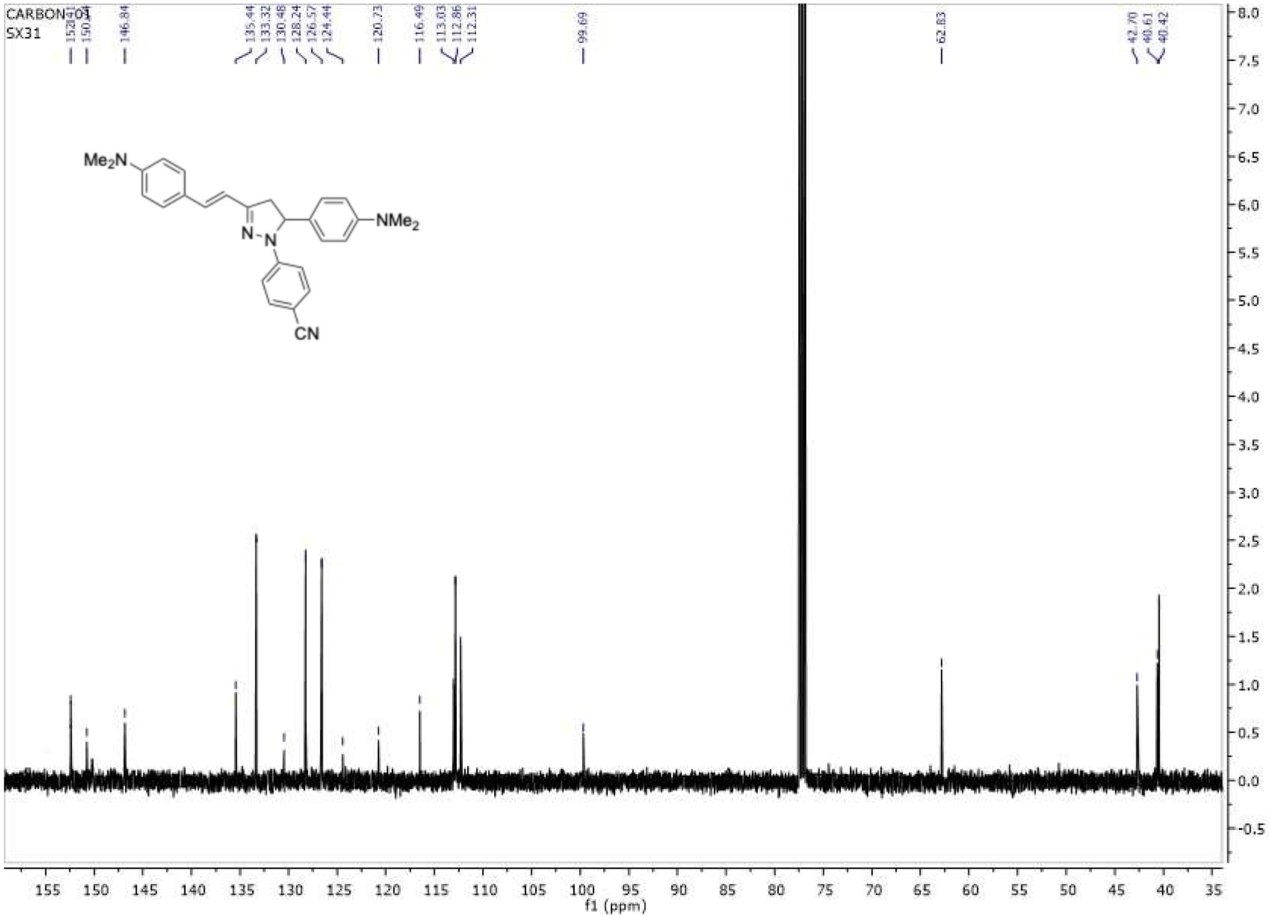

CARBON-01
SX31
152.41
150.8
146.84
135.44
133.32
130.48
128.24
126.57
124.44
120.73
116.49
113.03
112.86
112.31
99.69
62.83
42.70
40.61
40.42
Me2N
N
N
NMe2
CN
f1 (ppm)

SX31 corresponds to compound **4f**

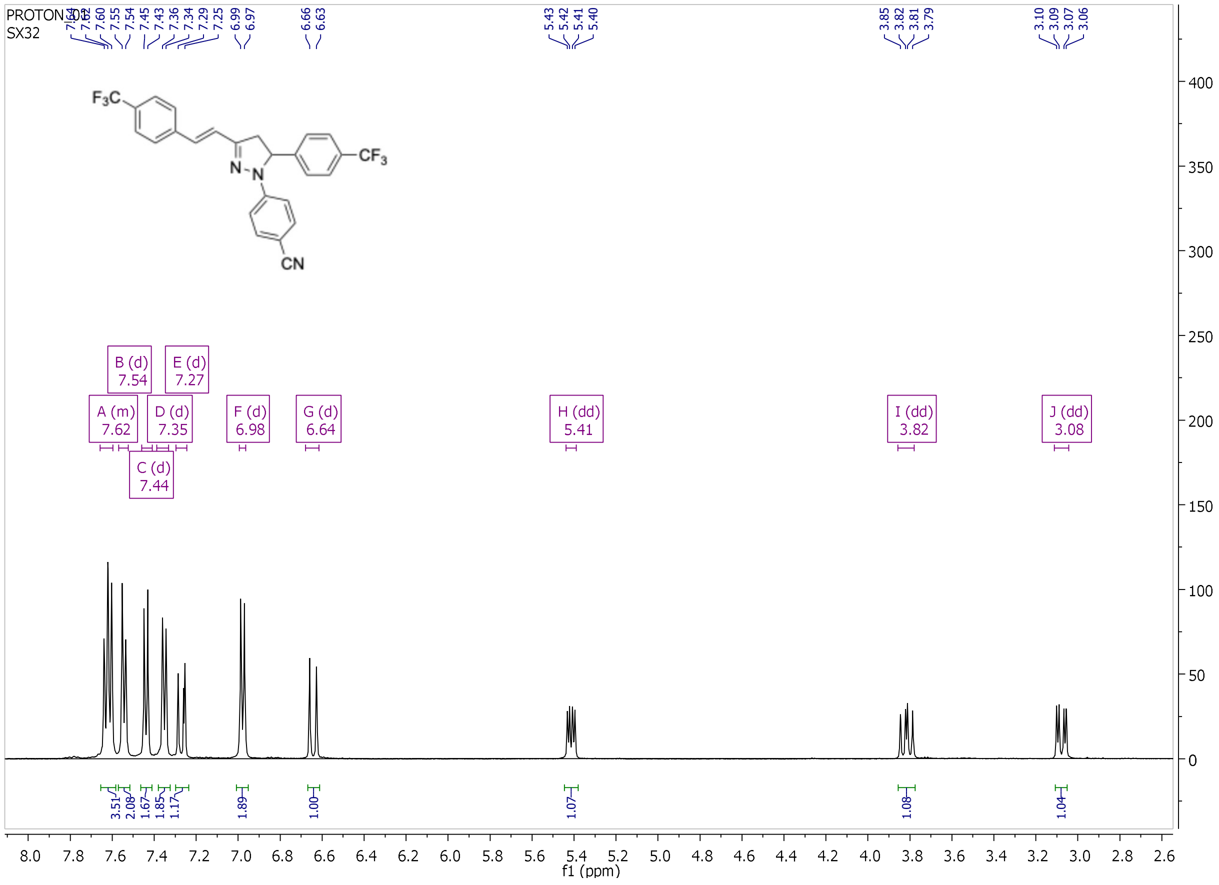

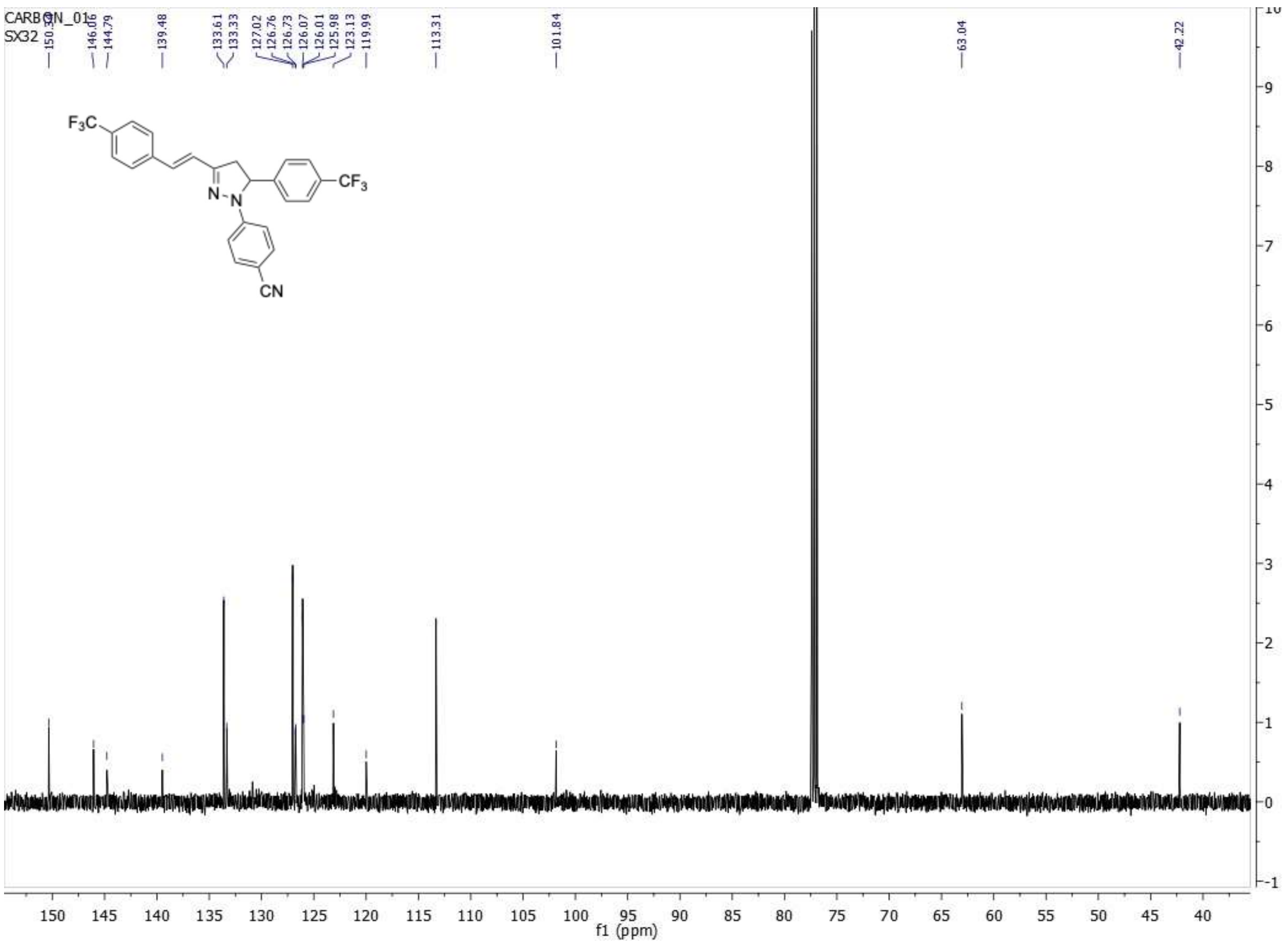

CARBON_01
SX32
150.39
146.06
144.79
139.48
133.61
133.33
127.02
126.76
126.73
126.07
126.01
125.98
123.13
119.99
113.31
101.84
63.04
42.22
F_3C
CF_3
N
N
CN
f1 (ppm)

E9_4 corresponds to compound **5b**

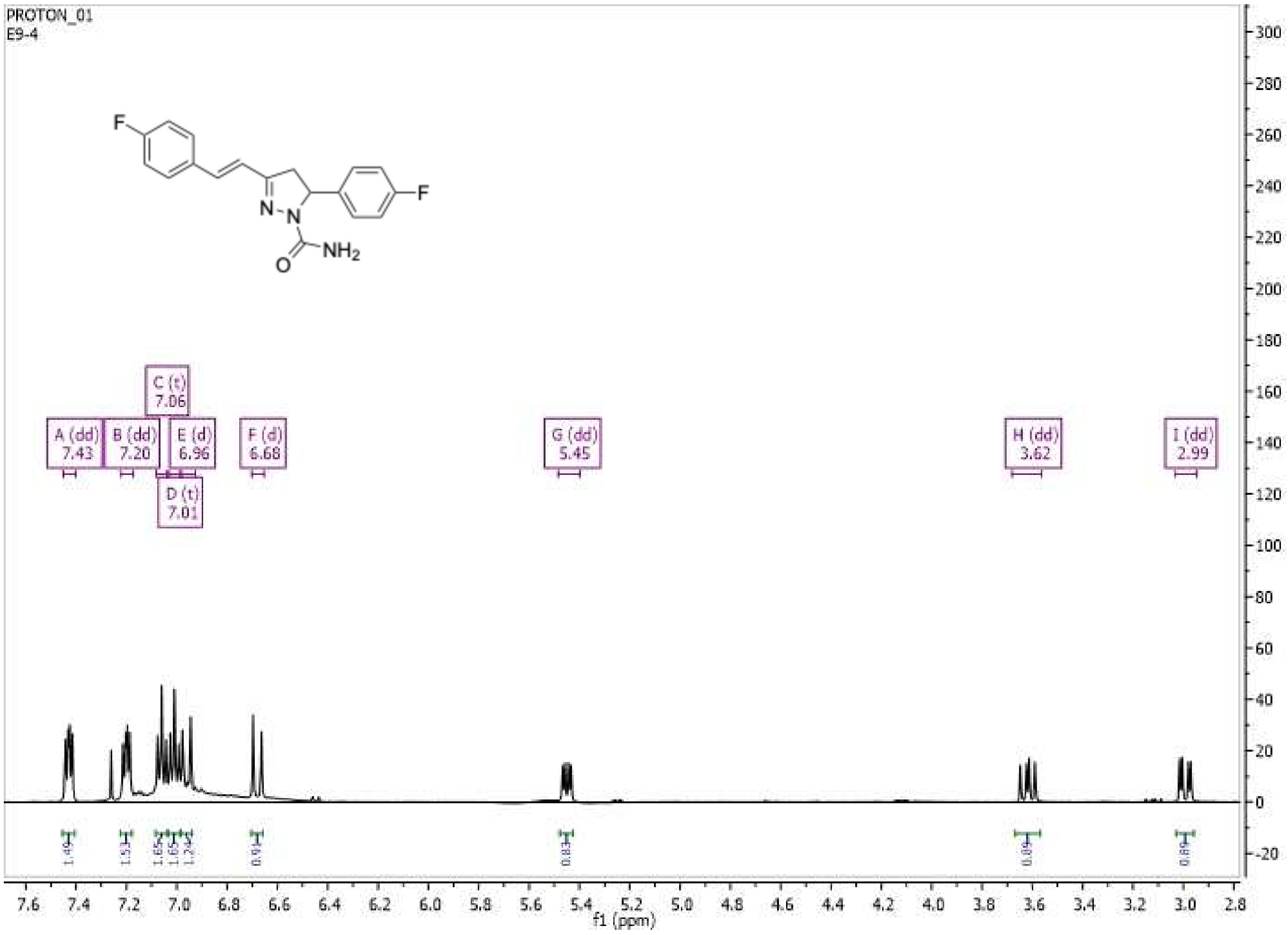

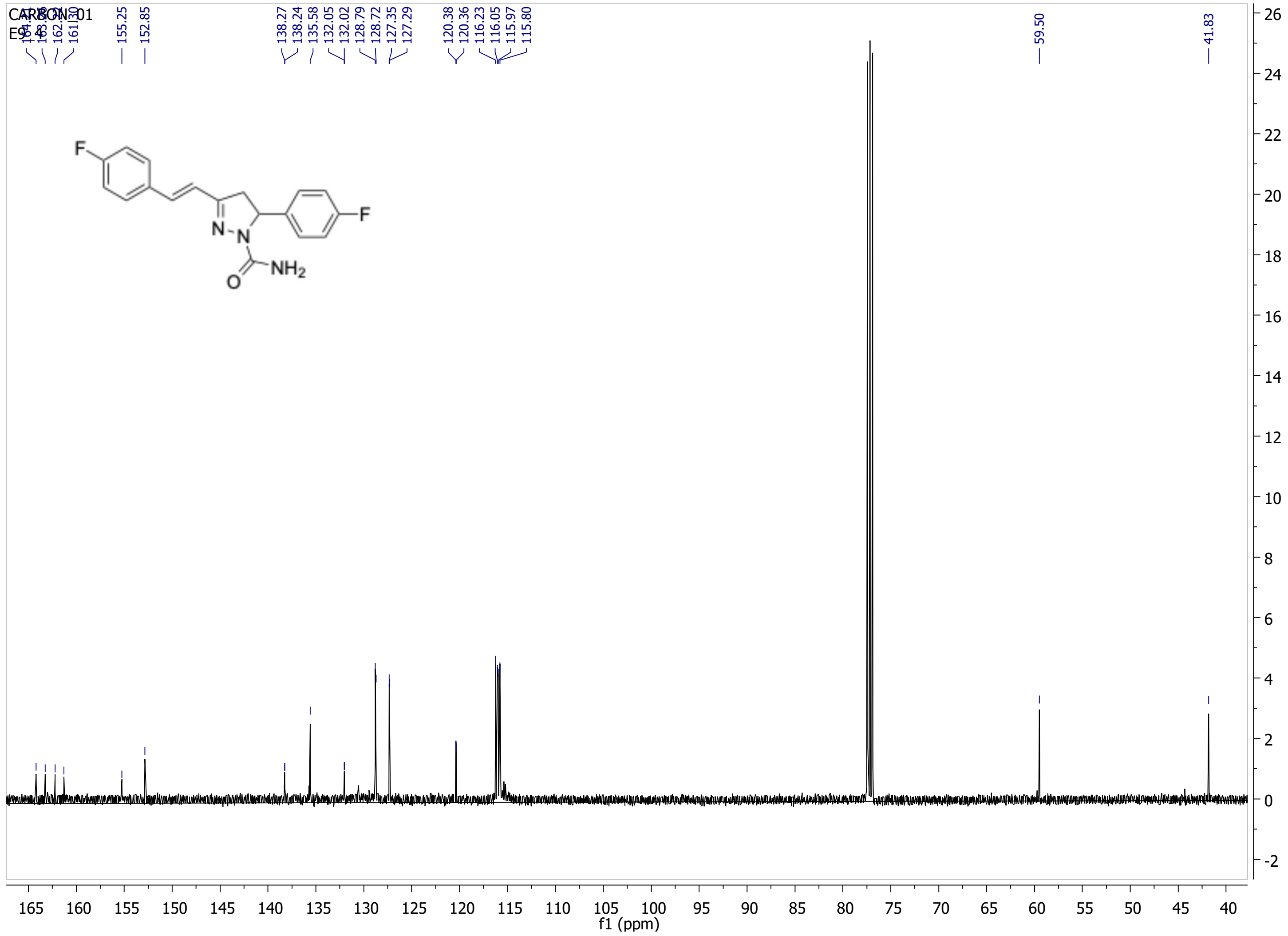

CARBON_01
E9_4
164.21
161.30
155.25
152.85
138.27
138.24
135.58
132.05
132.02
128.79
128.72
127.35
127.29
120.38
120.36
116.23
116.05
115.97
115.80
59.50
41.83
F
F
N
N
NH2
O
f1 (ppm)

E7 corresponds to compound **5c**

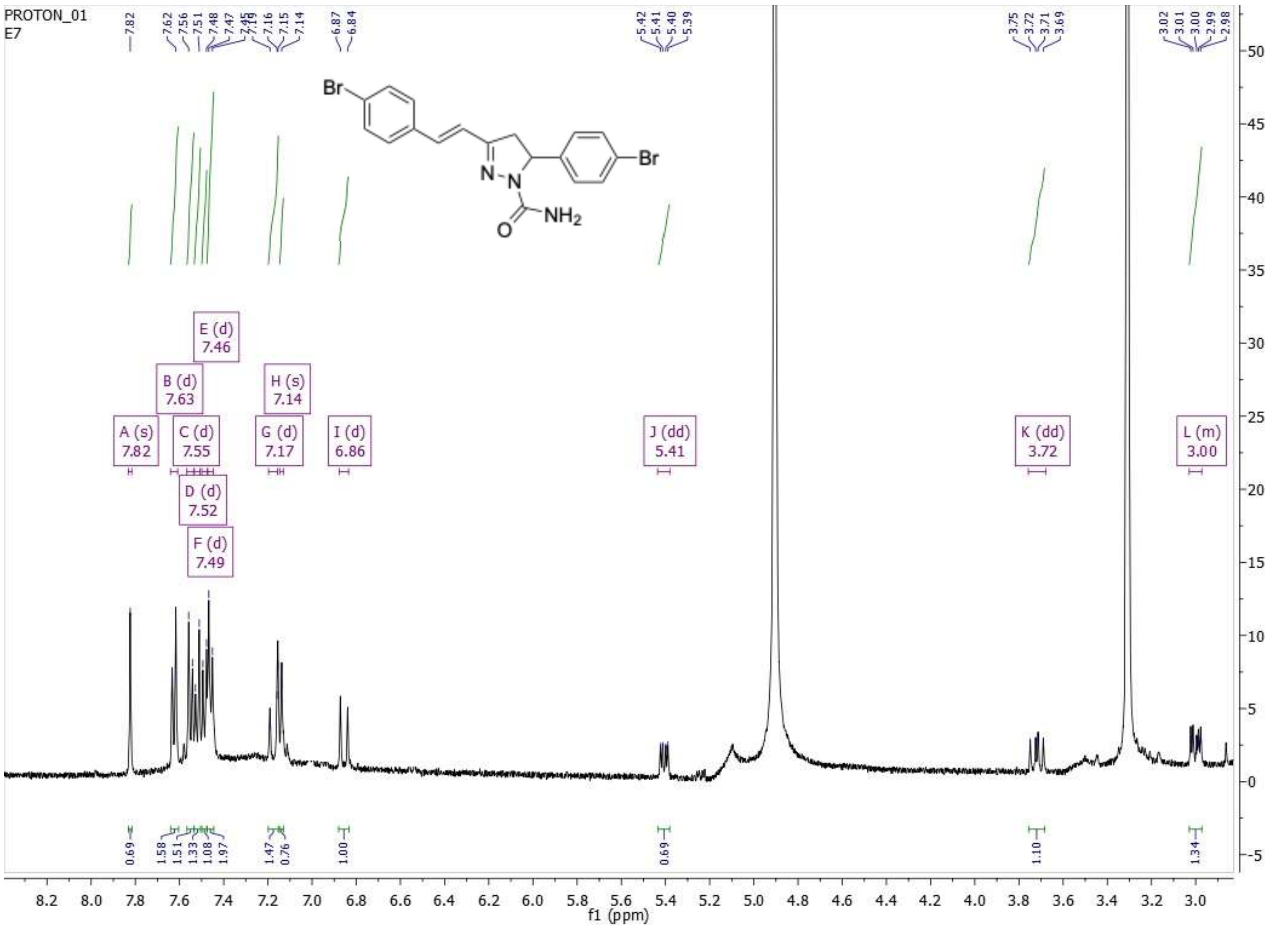

Supplement: Supplementary file 1 [file molecules-30-02224-s001.zip › molecules-3568162-supplementary.pdf]
